# Building better conversations: results of a community-based online health misinformation and motivational interviewing training program in Alaska

**DOI:** 10.1186/s12889-026-26611-1

**Published:** 2026-04-02

**Authors:** Drew B. Cameron, L. Grage, T. W. Hennessy, A. Cheng, M. Quinn, R. Van Wyck, J. A. Meyer, G. Garcia, J. C. Mapaye, K. Cueva

**Affiliations:** 1 Department of Health Policy and Management, Yale School of Public Health, Yale University, 60 College Street, New Haven, CT 06520 USA; 2 Information Insights, Fairbanks, AK USA; 3 Division of Population Health Sciences, University of Alaska Anchorage, Anchorage, AK USA; 4 Department of Social and Behavioral Sciences, Yale School of Public Health, Yale University, New Haven, CT USA; 5 L&M Policy Research, Washington, DC USA; 6 Department of Chronic Disease Epidemiology, Yale School of Public Health, Yale University, New Haven, CT USA; 7 Institute of Social and Economic Research, University of Alaska Anchorage, Anchorage, AK USA; 8 Department of Journalism and Public Communications, University of Alaska, Anchorage, Fine Arts Building, 3211 Providence Drive, Anchorage, AK 99508 USA

**Keywords:** COVID-19, Misinformation, Motivational interviewing, Pre-bunking, Role play

## Abstract

**Background:**

The COVID-19 pandemic has been accompanied by a proliferation of health misinformation, waning trust in health providers, and persistent vaccine hesitancy. To confront these challenges, a research team based in Alaska developed and pilot-tested an online training program called *Building Better Conversations*.

**Methods:**

Between July and September 2022, we delivered a total of nine trainings to 116 community members in Alaska, including health professionals, nurses, public health students, and public information officers, among others. Content included COVID-19 recommendations, health misinformation tactics, motivational interviewing strategies, and interactive role plays to practice addressing misinformation in realistic scenarios. Pre- and post-training surveys collected demographic data, impressions of the training, and changes in knowledge and self-efficacy. Qualitative interviews further explored attitudes towards the training and were examined using content and thematic analysis.

**Results:**

From July 2022 to April 2023, we collected 92 pre-training, 67 post-training, and 27 three-month follow-up surveys, and 38 in-depth interviews over two rounds. Participants expressed enthusiasm for the training, showing sustained improvements in misinformation recognition and self-efficacy identifying and discussing health information and misinformation, and talking with friends/family and others.

**Conclusions:**

This study highlights the potential of an educational program to improve knowledge and self-confidence to recognize and address health misinformation.

**Supplementary Information:**

The online version contains supplementary material available at 10.1186/s12889-026-26611-1.

## Introduction

Despite leading the country in COVID-19 vaccination early in the pandemic, the state of Alaska quickly fell behind national vaccination rates [1, 2]. As of August 2023 (the most recent data available), only 56.8% of eligible Alaskans had received a primary vaccine series [3], and only 15.6% had also received at least one booster dose [4]. Despite vaccination being one of the most effective public health tools to address and mitigate infectious disease, the COVID-19 era ushered in new challenges to vaccine acceptance [5]. Nationally, the 7 series childhood vaccination coverage rates dipped among children born in 2020 (from 75.9% coverage at 35 months of children born in 2012, to 73.9% coverage for children born in 2020) [6], and these rates remained below the national average in Alaska following the COVID-19 pandemic [7].

These trends are fueled in large part by a health misinformation and disinformation environment that has proliferated with deadly consequences [8]. The study of misinformation, and indeed its very definition, are the subject of ongoing research [9–11]. The American Psychological Association defines *misinformation* as “false or inaccurate information” and *disinformation* as “false information which is deliberately intended to mislead.” [12] Herein we focus on specific strategies used to spread mis- and disinformation (e.g., the use of emotional language, false dichotomies, and fake experts) [13–15]. Mis- and disinformation can undermine trust in healthcare professionals, public health organizations, and governmental agencies [16–22], and can lead to risky health behaviors and higher rates of death [16, 23, 24]. Effective strategies to address prolific health misinformation and disinformation are crucial to prevent unnecessary morbidity and mortality. Starting in 2020, a team based at the University of Alaska, Anchorage (UAA) began coordinated efforts to monitor and mitigate the COVID-19 pandemic as Alaska’s COVID-19 vaccination rates plateaued amidst growing mis- and disinformation [25–28]. The team consequently pivoted to engage in work addressing health misinformation. One branch of this work included developing and piloting a community-based online training called *Health Misinformation in Alaska: Building Better Conversations.*

## Background

### Developing the training

The training was developed collaboratively by faculty, researchers, and graduate students from UAA and the Yale School of Public Health. The design was informed by several evidence-based strategies to insulate/inoculate against health misinformation [13–15, 29–32], as well as guidance and materials from the World Health Organization [33], the United Nations Children’s Fund [34], the Centers for Disease Control and Prevention (CDC) [35, 36], and the Surgeon General’s advisory on building a health information environment [37]. These included providing education on common tactics employed in the spread of mis- and disinformation and how to talk to friends and family affected by health misinformation. Further evidence from Alaska also suggests vaccine confidence interventions should specifically address intrinsic motivation [38]. Thus, the training integrated elements of motivational interviewing, an evidence-based method to facilitate positive health behavior change [39–41], increasingly leveraged in community interventions [42–44]. At the time of design, motivational interviewing was considered a potentially powerful yet understudied tool to address COVID-19 vaccine hesitancy [45–48].

A key element of the training also included the opportunity for participants to engage in role plays of realistic scenarios where they may encounter common COVID-19 health misinformation. Role play is a common teaching strategy that allows a participant to “try on” the words, thoughts, and feelings of a different perspective [49], as well as practice responding to a potentially challenging situation in a well-facilitated environment. Previous studies on the use of role play in health-related settings suggests it is widely seen as beneficial by participants and can improve their self-efficacy to address health misinformation [50, 51].

**Fig. 1:**
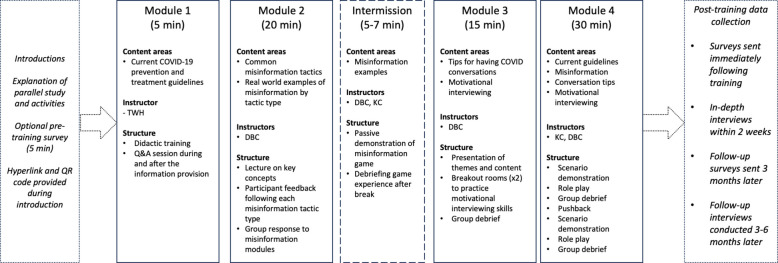
Structure of the training

The structure of the training, highlighted in Fig. 1, included:

- Trainer introductions and time to take an optional pre-training survey.
- Current COVID-19 information, trends, and recommendations.
- Definitions of misinformation and disinformation and common examples of tactics being used on social media in Alaska.
- An intermission to play the online game *Go Viral*, an evidence-based psychological intervention to help people identify and resist health misinformation [13]
- Strategies to address health misinformation.
  - ◦ Skills adapted from the Office of the Surgeon General’s community toolkit for addressing health misinformation [52].
  - ◦ Motivational interviewing strategies using the OARS (Open-ended questions, Affirmations, Reflections, Summaries) method [53].
- Role plays to address health misinformation in realistic settings.
  - ◦ Demonstration of a role play scenario by facilitators.
  - ◦ Participant role play practice in pairs.
    - ▪ Group debrief.
  - ◦ Information on countering resistance.
  - ◦ A demonstration of a second role play, where the misinformed individual was more resistant to engaging in conversation.
  - ◦ Participant role play of the second scenario in pairs.
    - ▪ Group debrief.
- Invitation to take a post-training survey and distribution of handouts to participants.

**Fig. 2:**
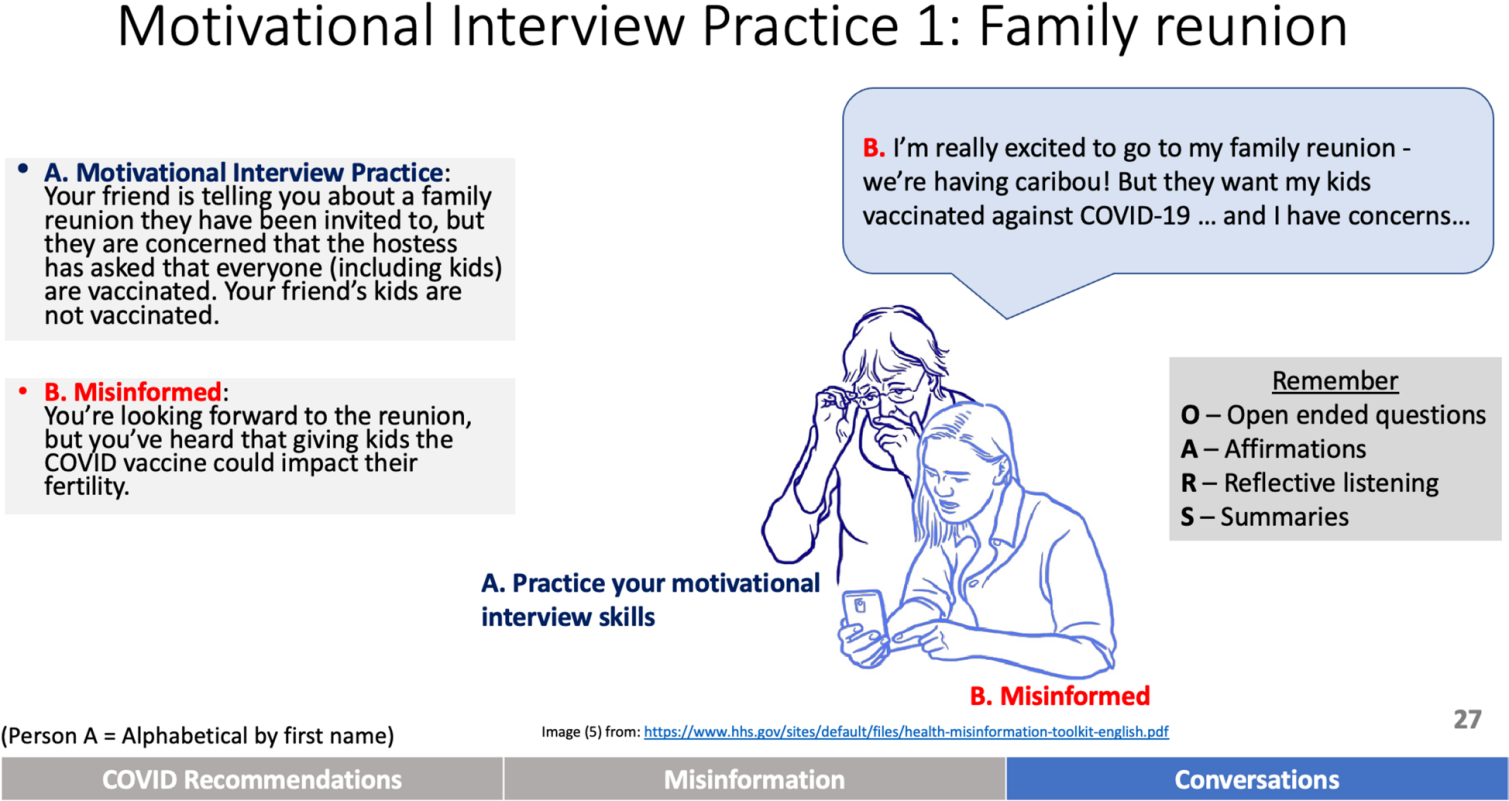
Example of a motivational interviewing role play scenario

The training was delivered by individuals from, and well-connected within, Alaska. Dr. Tom Hennessy, an infectious disease epidemiologist and former director of the CDC Arctic Investigations Program who was instrumental in organizing the Alaska response to COVID-19, presented current COVID-19 information, trends and recommendations. Didactic training modules (i.e., teacher-centered instruction conveying specific content or skills through lectures, presentations, etc.) on misinformation identification, common tactics, and responding to health misinformation were presented by Dr. Drew Cameron who was born and raised in Fairbanks, Alaska. The interactive portion of the training, including demonstrations and opportunities for learners to engage in role playing (e.g., Fig. 2 shows a role play prompt for practicing motivational interviewing), and debriefs, was facilitated by Dr. Katie Cueva, who was raised and resides in Anchorage, Alaska. Additional training support and recruitment was provided by individuals living in Alaska. Appendix 1 contains the full training PowerPoint and handout with links to recorded content.

### Focus region and timeline

**Fig. 3:**
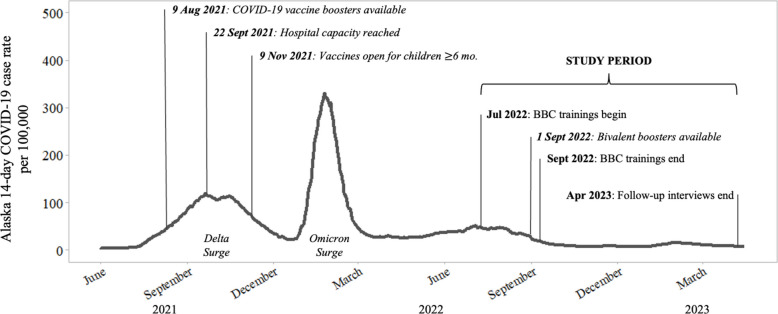
Training and study period within COVID-19 pandemic timeline

Using data from the state vaccine dashboard [54], and the results of statewide surveys [38, 55, 56], boroughs (county equivalents) with lower rates of COVID-19 vaccination were identified. In July 2022, the Fairbanks North Star Borough had a population of about 96,922 and 57% of residents had received an initial COVID-19 vaccination, not including data from the DOD/VA [54, 57]. Figure 3 situates the timing of the *Building Better Conversations* training as well as key study activities within the timeline of the COVID-19 pandemic, with the 14-day COVID-19 case rate on the y-axis and major pandemic events within Alaska italicized for clarity.

### Training participant recruitment

Training participant recruitment took place between 1 July and 20 September 2022. Personal connections allowed for targeted outreach, focusing on potentially trusted sources of health information in Fairbanks. Recruitment for trainings was done through word of mouth and emails to targeted individuals and listservs that included a flyer with training dates. Individuals were also targeted through professional and social networks, statewide announcements through the Alaska Health Education Center (AHEC) listserv, and a training offered to incoming Master of Public Health (MPH) students at UAA. In total, 116 training participants were recruited, including 76 (65.5%) through targeted outreach to specific groups, 10 (8.6%) via the AHEC listserv, and 30 (25.9%) through the incoming MPH student training. Trainees were invited, but not required, to participate in the research study, which we explain in more detail in the Methods section.

## Methods

### Study recruitment

At the start of each training, participants were invited to take part in an optional research study and take a pre-training survey. Anyone who started the survey was later invited by email to complete a post-training survey, a three-month follow-up survey, and up to two in-depth interviews (see Appendix 2 for full survey instruments). Post-training survey invitations were sent 3-h after initiating the pre-training survey. Up to three post-training interview recruitment emails were sent to participants who completed the pre-training survey. Additional invitations were sent via email three months after the training for a three-month follow-up survey and three to six months after the training for follow-up interviews. Incentives included gift cards to a local grocery store valued at $20 for each post-training or three-month follow-up survey, and $45 for each in-depth interview completed. All subjects provided informed consent to participate in surveys and recorded in-depth interviews.

### Data collection

**Fig. 4:**
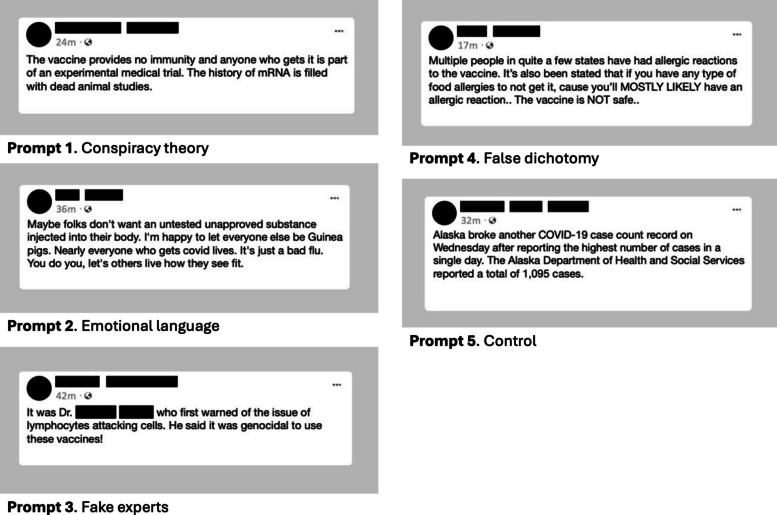
Example survey prompts on misinformation

Surveys in Qualtrics were designed to measure changes in knowledge, attitudes, and intentions regarding health misinformation and engagement with misinformation, as well as impressions of the training. The pre-training survey included questions on demographics, vaccination status, and pre-training expectations. Post-training surveys included personal ratings of training components, open-ended questions about likes and dislikes from the training, and suggestions for improvement. Surveys also contained prompts modified from prior misinformation identification trials [13–15] asking respondents to rate how “*manipulative*” they found a series of real Facebook posts using misinformation strategies including *conspiracy theories*, *emotional language*, *fake experts*, and *false dichotomies* (see Fig. 4), as well as a control post containing factual COVID-19 information. Due to a coding error, only 3 out of 4 misinformation prompts were randomly displayed to respondents during both pre- and post-training surveys rather than all 4 prompts, as intended. Surveys also included questions asking respondents to rate their confidence (self-efficacy) in their ability “*to identify accurate information about COVID-19,*” “*identify misinformation,*” as well as their ability to have difficult conversations “*with friends/family*” and “*others.*”

In-depth interviews were conducted via Zoom. Interview guides were designed by adapting selected questions taken from the Consolidated Framework for Implementation Research (CFIR) [58, 59]. Most questions were drawn from within the CFIR domains: *Intervention Characteristics* (e.g., intervention source, evidence strength and quality, adaptability, design quality and packaging), *inner setting* (e.g., tension for change, goals and feedback), and *characteristics of individuals* (e.g., knowledge and beliefs, self-efficacy, individual stages of change). Modified interview questions were then re-organized into new categories. Post-training interviews covered respondents’ 1) professional background and roles, 2) reasons for taking the training, the role of the training in 3) building misinformation confidence/skills, 4) building motivational interviewing confidence/skills, 5) future plans/intentions, and 6) potential for reach. Follow-up interview topics expanded into 7) experiences putting the training into practice, training topics that were 8) easier, or 9) harder to remember, community members with whom respondents 10) shared, 11) applied, or 12) planned to apply lessons from the trainings, and 13) future avenues for support. In-depth interview guides are available in Appendix 3.

### Data analysis

This study used a mixed-methods approach. Quantitative and qualitative data from surveys were analyzed using Stata v18 and included descriptive statistics, frequency distributions and mean and ANOVA comparisons. In-depth interview transcripts and open-ended survey responses were analyzed using content and thematic analyses. For in-depth interviews, a three-person team (DBC, LG, AC) independently coded 38 transcripts using NVivo (v.17.1). Preliminary “meta categories,” “parent” and “baby” codes were established to classify themes. After reconciling coding differences to attain consensus on categories, a second double-coding round was conducted. Final codebooks are available in Appendix 3.

## Findings

### Trainings and demographics

**Fig. 5:**
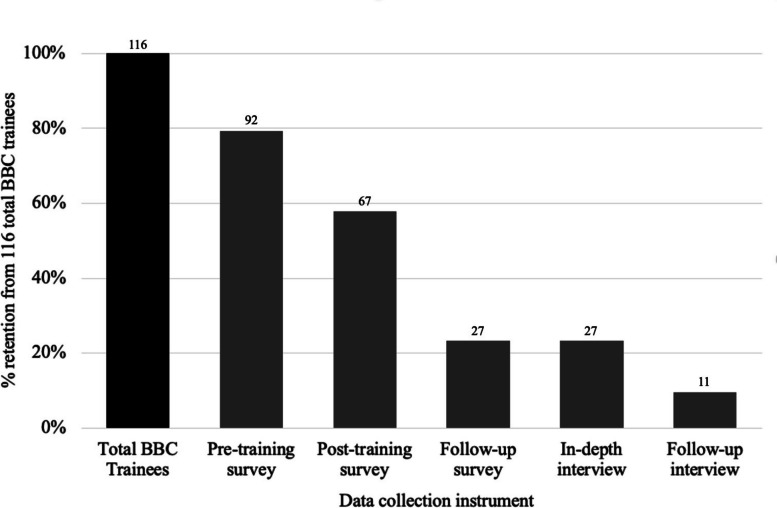
Retention of study participants

Figure 5 characterizes participant retention by study instrument. Of 116 trainees from the nine training sessions taking place from July through September 2022, 92 (79%) took the pre-training survey, 67 (58%) completed the post-training survey and 27 (23%) participated in post-training interviews. Later three-month follow-up surveys were completed by 26 trainees (22%), while 11 (10%) sat for follow-up interviews. Pre-training surveys took a median of 5.5 min. Participants completed post-training surveys an average of 1.3 days after the training (range: 0–17 days), taking a median of 12.5 min. Post-training interviews took place an average of 12 days after each training (range: 2–28 days) and lasted 40 min to one hour. Three-month follow-up surveys were completed an average of three months after trainings (range: 70–125 days), taking a median of 13.8 min to complete. Follow-up interviews took place an average of six months after trainings (range: 81–250 days) and lasted between 40 min to one hour.

Table 1 describes demographic characteristics of pre-training survey participants. Most were female (88%), White (76%), from the Fairbanks North Star Borough (57%) or the Anchorage municipality (21%). Most worked as health professionals (49%) in education (20%) or in public affairs (11%). Although most (73%) were vaccinated and boosted with a COVID-19 vaccine, about a quarter (26%) were either unvaccinated or had not received a booster. Participant demographics were compared by data collection instrument using chi-squared tests (unadjusted for multiple-hypothesis testing). Compared to those who completed the pre-training survey, post-training interview respondents were less likely to be health professionals, and more likely to be retirees and public affairs/information officers (*p* = 0.046). Compared to the pre-training survey, participants in the follow-up interviews were more likely to be white (*p* = 0.048) and male (*p* = 0.026).

**Table 1:** Study participant demographics

Participant characteristics	*Pre-training survey (n)*	*%*
Gender		
Male	8	*9%*
Female	81	*88%*
Other or no answer	3	*3%*
Age		
18–29	16	*17%*
30–39	23	*25%*
40–49	16	*17%*
50–59	14	*15%*
60–69	12	*13%*
70 <	10	*11%*
No Answer	1	*1%*
Race/Ethnicity^a^		
White	70	*76%*
Alaska Native/American Indian	11	*12%*
Asian	5	*5%*
Latinx	5	*5%*
Other	6	*7%*
No Answer	3	*3%*
Region		
Fairbanks North Star Borough	52	*57%*
Anchorage	19	*21%*
Other Alaskan borough	15	*16%*
No answer/Other US state/territory	6	*7%*
Educational achievement		
Associate's degree or less	23	*25%*
Bachelor's degree	41	*45%*
Master's degree or more	26	*28%*
No answer	2	*2%*
Primary vocation		
Admin	4	*4%*
Education	18	*20%*
Health professional	45	*49%*
Public affairs/public information officer	10	*11%*
Retired or self-employed	7	*8%*
No answer	8	*9%*
Salary		
$30,000 and under	9	*10%*
$30,001—$50,000	17	*18%*
$50,001—$70,000	18	*20%*
$70,001—$90,000	19	*21%*
$90,001 <	22	*24%*
No answer	7	*8%*
Vaccination Status		
Vaccinated and boosted	67	*73%*
Only initial vaccine or series (no booster)	14	*15%*
Unvaccinated or only partial vaccination	10	*11%*
No answer	1	*1%*

^a^Participants selected all racial/ethnic groups that applied; Other includes Black, Pacific Islander, Filipino, and Middle Eastern

### Trainee expectations

#### Pre- and post-training survey findings

Table 2 outlines participant expectations and whether they were met. Pre-training survey respondents were asked “*What do you hope to achieve from this training?*” Overall, 55% had communication goals like increasing confidence/skills, followed by misinformation-related goals (34%) like combatting or identifying health misinformation, COVID-19 information goals (27%), and general interest (19%). Most respondents (68%) in non-healthcare professions mentioned a communication-related goal compared to only 42% of healthcare workers (*p* = 0.013, chi-squared test). When asked “*did you learn what you hoped from the training?*”, most of the post-training survey respondents (63%) were positive. The remaining answered “*somewhat*” (9%), “*no*” (6%), or offered some additional comments (6%).

**Table 2:** BBC training expectations: Pre- and post-training survey open-ended responses

		*n*	%
Pre-training survey question: "What do you hope to achieve from this training?" (*n*=92)			
No response/unsure		7	7.6%
General interest^a^		17	18.5%
COVID-19 information goals		25	27.2%
*spread correct information*		20	21.7%
*gain current info/recommendations*		6	6.5%
Misinformation-related goals		31	33.7%
*combat misinformation*		16	17.4%
*identify/understand misinformation*		14	15.2%
*distinguish between info and misinformation*		9	9.8%
Communication skills-related goals		51	55.4%
*increased confidence/skills*		32	34.8%
*...with misinformed people*		19	20.7%
*...with specific clients/communities*		9	9.8%
*...with friends/family*		7	7.6%
Total		92	
Post-training survey question: "Did you learn what you hoped from this training?" (*n*=67)			
No response		11	16.4%
Yes		42	62.7%
Somewhat		6	9.0%
No		4	6.0%
Other		4	6.0%
Total		67	

^a^General interest responses include "anything," "everything," "general skill building," and "improving public health behaviors"

#### Post-training interview findings

A majority of the 27 post-training interviewees noted specific instances of having previously encountered health misinformation, 19 (70%) in their personal and 16 (59%) in their professional lives. In total, 21 interviewees (78%) encountered health misinformation about vaccines, followed by 13 (48%) who cited masks, 12 (44%) the government, ten (37%) about the SARS-CoV-2 virus, seven (26%) regarding general confusion about COVID-19 guidelines, and six (23%) about treatments for COVID-19. Of all post-training interviewees, 16 (59%) said they attended the training to develop new skills, while 13 (48%) expressed general curiosity or support for the topic as a reason for attending. As one participant shared:

“I was interested in this to see if there were any new techniques to counter some of the misinformation that people have and help them make decisions that they're comfortable with.”

Nine interviewees (33%) noted either social pressure or a personal connection as the main reason they attended, and four (15%) said the training was required.

### Trainee satisfaction

#### Post-training survey findings

Post-training surveys were completed by 67 participants. Respondents rated both the quality and the informativeness of the training modules very highly. Among post-training survey respondents, 82% answered the open-ended question “*What did you like about this training?*” Table 3 displays results, which could be coded into multiple categories. Respondents commented on the training content (64%) and format (40%). In content, the largest share liked the role plays (27%), didactic misinformation content (22%), motivational interviewing (21%), general content (16%), and communication strategies (13%). Format feedback focused on general format/presentation (12%), interactivity (12%), professionalism of the training team (12%), and use and relevance of examples (10%).

**Table 3:** Open ended responses to "What did you like about the training?"

Topic area	Specific comment	*n*	%
No response		12	18%
Nothing - respondent didn't like anything		0	0%
Format		27	40%
	*General format/presentation*	8	12%
	*Interactivity*	8	12%
	*Professionalism of training team*	8	12%
	*Use and relevance of examples*	7	10%
	*Feeling of openess, welcoming, safety*	4	6%
	*Other*	5	7%
Content		43	64%
	*Role play*	18	27%
	*Didactic misinformation content*	15	22%
	*Motivational interviewing*	14	21%
	*General content*	11	16%
	*Communication strategies*	9	13%
Other		4	6%
Total		67	100

A total of 55 respondents provided any response; Responses could be coded into multiple categories

Table 4 summarizes feedback from 50 respondents (75% of all post-survey participants) to the question “*What did you dislike about this training?*” Again, responses could be coded into multiple categories. Of all post-training respondents, 16% felt they wanted more time (either overall or for role plays), 13% wanted more and harder scenarios to practice, 10% requested real-life scenarios from other trainees working in healthcare, 10% requested information on provider burnout, and 10% offered suggested improvements to the structure of the role plays. An additional 9% said they liked everything and 7% cited technical difficulties.

**Table 4:** Open ended responses to "What did you dislike about the training?"

Topic area	Specific comment	*n*	%
No response		17	25%
Nothing - respondent liked everything		6	9%
Response unrelated to training		3	4%
Format		32	48%
	*Not enough time*	11	16%
	*Role play format suggestions*	7	10%
	*Technical difficulties*	5	7%
	*Not interactive enough*	3	4%
	*Other*	18	27%
Content		15	22%
	*More and harder role play scenarios*^a^	9	13%
	*COVID-19 recommendations unnecessary*	3	4%
	*More misinformation content*	3	4%
	*Other*	4	6%
Total		67	100

A total of 50 respondents provided any response; Responses could be coded into multiple categories

^a^Respondent comments included wanting more advice and examples on difficult interactions, including real life examples from healthcare participants and addressing empathy and burnout among healthcare workers

#### Post-training interview findings

Out of 27 post-training interviewees, 17 (63%) shared general feelings about the training during interviews. Of these, 13 (48%) gave positive feedback including appreciating the clear presentation of information, organization, and contextual relevance.

“I think the presentation of information was clear. It was organized, and it provided the right amount of context, so recognizing what's the deal with COVID-19 in Alaska right now and then getting into types of misinformation, disinformation, and then jumping—I think I didn't leave and think, ‘Oh, there's anything missing.’”

A total of 23 post-training interviewees (85%) shared specific examples of what parts of the training they liked. Of these, the most common theme came from 20 respondents (87%) who liked the role-playing.

“As silly as they are sometimes, role-playing is necessary. Especially if you can kind of rehearse a conversation to have in public or even to a family member. I think that's the biggest takeaway.”

Other themes included 11 interviewees (48%) liking the resources shared, which were described by one respondent as “right on track, consistent [and] helpful,” nine (39%) liked the information shared about how to respond to health misinformation using motivational interviewing strategies, and eight (35%) liked the overall structure of the program.

Only five interviewees shared that they disliked something about the training. Of these, three (60%) said they didn’t like something about the structure, organization, or format of the training, such as the scheduled break, while two (40%) shared that they didn’t like role-playing activities but conceded that they were nonetheless important elements to practice.

A total of 25 interviewees (93%) shared specific suggestions to improve the training. Of these, 22 (88%) suggested modifying existing components and wanted additional context and examples, or more time for the training.

“I would have liked a little more time, perhaps seeing examples and being able to pinpoint what misinformation strategies are being used here … maybe just a little, I don’t know, minute or two-minute-long activity of like, ‘Okay, let’s look at this one. Let’s see what strategies they’re using.’ Just more audience interaction.”

A total of 15 participants (60%) suggested the inclusion of new information, and one (4%) suggested removing content.

### Training content

#### Post-training survey findings

Table 5 summarizes trainee misinformation recognition, and self-efficacy measures, comparing within-subject ratings between pre- and post-training surveys using paired *t*-tests. When asked how “*manipulative*” social media posts were (see Fig. 3), ratings increased for the post featuring *emotional language* (*p* = 0.005) and on average (*p* = 0.039). Participant self-efficacy improved from pre- to post-training for confidence to identify accurate information (*p* = 0.006), misinformation (*p* = 0.001), and to talk about misinformation with friends/family (*p* < 0.001) and others (*p* < 0.001).

**Table 5:** Misinformation identification ability and self-efficacy at pre- versus post-training survey - paired t-tests

Assessment tool	Pre-training survey	Post-training survey	△	*p-value*		*n*
Misinformation prompts, mean ratings (scale 0-7)						
conspiracy theory	5.9	5.6	-0.2	0.212		39
sd	1.6	2.0				
emotional language	5.5	6.3	0.8	0.005	***	38
sd	1.8	0.8				
fake experts	5.9	6.0	0.1	0.782		34
sd	1.3	1.6				
false dichotomy	5.7	6.1	0.4	0.079	*	38
sd	1.6	1.1				
average misinformation rating	5.7	6.0	0.3	0.039	**	65
sd	1.3	1.4				
control	2.1	2.2	0.0	0.803		65
sd	1.4	1.7				
Self-efficacy (confidence) prompts, mean ratings (scale 1-10)						
identifying accurate information	8.5	8.8	0.4	0.006	***	65
sd	1.8	1.6				
identifying misinformation	8.0	8.7	0.6	0.001	***	61
sd	1.7	1.2				
talking with friends / family	7.7	8.4	0.7	<0.001	***	64
sd	2.0	1.6				
talking with others	7.1	8.2	1.0	<0.001	***	62
sd	2.3	1.6				

* *p*<0.1, ** *p*<0.05, *** *p*<0.01; Delta signifies mean within-participant change from pre- to post-training survey

Table 6 outlines the difference in outcomes between participants by vaccination status using Student’s *t*-tests. In the pre-training survey, self-assessed confidence to identify misinformation was 1.2 points higher on average (*p* = 0.001) among fully vaccinated and boosted respondents (*n* = 67) compared to those who had not been vaccinated (*n* = 24). In the post-training survey, that difference decreased on average, but was only significant for *emotional language* (*p* = 0.045). For self-efficacy, confidence to identify accurate health information was 1.3 points higher among those who were fully vaccinated and boosted at pre-training (*p* = 0.016) and post-training (*p* = 0.009).

**Table 6:** Misinformation identification ability and self-efficacy at baseline and post-training survey by vaccination status - Student's t-tests

Assessment tool	PRE-TRAINING SURVEY					*n*	POST-TRAINING SURVEY					*n*
	Vaccinated and boosted (mean)	Unvaccinated or not boosted (mean)	Student's t-tests				Vaccinated and boosted (mean)	Unvaccinated or not boosted (mean)	Student's t-tests			
			diff.	*p*-value					diff.	*p*-value		
Misinformation prompts mean ratings (scale 0-7)												
conspiracy theory	6.1	5.0	-1.1	0.027	**	55	6.0	5.3	-0.7	0.257		47
sd	1.3	1.9					1.7	2.3				
emotional language	5.8	4.1	-1.7	0.003	***	48	6.3	5.2	-1.0	0.045	**	50
sd	1.6	1.4					1.3	1.9				
fake experts	6.0	4.9	-1.1	0.039	**	48	6.1	5.2	-0.9	0.086	*	48
sd	1.3	1.7					1.2	2.2				
false dichotomy	5.9	4.8	-1.0	0.037	**	50	6.2	5.9	-0.4	0.374		50
sd	1.4	1.7					1.0	1.3				
Average Misinformation Rating	5.9	4.7	-1.2	0.001	***	67	6.2	5.4	-0.8	0.066	*	65
sd	1.2	1.3					1.2	1.9				
control	2.1	2.4	0.3	0.521		67	2.1	2.3	0.2	0.707		65
sd	1.5	1.3					1.7	1.6				
Self-effacacy (confidence) prompts mean ratings (scale 1-10)												
identifying accurate info	8.7	7.4	-1.3	0.016	**	67	9.1	7.8	-1.3	0.009	***	65
sd	1.4	2.7					1.0	3.0				
identifying misinfo	8.2	7.4	-0.7	0.145		64	8.5	8.5	0.0	0.928		64
sd	1.5	2.2					1.3	1.2				
talking with friends / family	7.8	7.5	-0.3	0.677		66	8.5	7.9	-0.6	0.234		65
sd	1.9	2.2					1.5	2.2				
talking with others	7.3	6.4	-0.9	0.189		65	8.3	7.5	-0.8	0.119		64
sd	2.1	2.9					1.5	2.1				

* *p*<0.1, ** *p*<0.05, *** *p*<0.01; Due to a coding error, only 3 out of 4 misinformation prompts (i.e., conspiracy theory; emotional language; fake experts; false dichotomy) were randomly displayed to respondents during both pre-training and post-training surveys, while the control prompt was delivered to all respondents

#### Post-training interview findings

Post-training interviews illustrated modest participant gains in misinformation recognition, and improvements in self-efficacy. As one participant shared:

“I do feel more prepared to be able to share the information in a confident way, like just with my colleagues and our staff here.”

Of the 27 interviewees, 16 (60%) felt they had previous knowledge reinforced, while 14 (51%) felt they had learned new ways of applying that knowledge towards misinformation. This sentiment was well captured by one participant:

“I mean, I think genuinely it was helpful. I think that a lot of the things that we went through I was doing anyways. Just because I had to have a lot of conversations. The methods of motivational interviewing or empathizing with the person. All of those are things that I just do because I'm like, ‘I want us to be friends. I don't want this to get crazy.’”

When asked their feelings about using motivational interviewing tactics in the future, 25 interviewees (93%) provided feedback. Of these, 22 (88%) indicated they felt positively about using those skills in the future, while 15 (60%) also indicated some neutral or uncertain feelings about applying those skills in their lives. Four interviewees (16%) expressed a negative outlook. One participant captured this mixed feeling succinctly:

“I don’t know. I’m not sure what the right word would be, resolve, encouragement. I don’t know. A concept that maybe it’s not [a] completely lost course to try and help people get the right information, I guess. It can be pretty overwhelming sometimes and especially if it’s these waves, constant waves of stuff that you see.”

### Sustainability and Reach

#### Three-month follow-up survey findings

Table 7 examines persistence of outcomes among 25 respondents who completed all three surveys. Misinformation recognition for *emotional language* increased significantly from pre- to post-training survey (*p* = 0.018) without significant reversions to the pre-training mean at three months post-training. There were significant improvements in two of four self-efficacy ratings from pre- to post-training survey with no sign of waning at three months post-training. The self-efficacy measure for *having conversations with other members of one’s community* increased significantly in both post- and three-month follow-up survey (repeated measures ANOVA, *p* = 0.002), with results robust to Box's conservative epsilon (*p* = 0.014). When asked if they used motivational interviewing skills in the time since trainings ended, 74% responded affirmatively. Of these, 90% said that the conversation went “*better than it could have,*” and that their relationships after these encounters were the same or better than before. When asked if they had shared anything they learned from the training, 81% had shared with at least one other person like a co-worker (58%), family member (42%), friend (38%), or others including clients, neighbors, and other community members (31%). Under half of respondents (41%) indicated they would be interested in a refresher training.

**Table 7:** Sustainability of misinformation identification ability and self-efficacy at pre- vs. post- vs. follow-up training surveys - Paired t-tests and repeated measures ANOVA

Assessment tool	Pre-training survey (mean)	Post-training survey (mean)	Follow-up survey (mean)	Pre- vs. post- paired t-test		ANOVA (pre-, post-, follow-up repeated measures)				n
				*p*-value		F-test	*p*-value		n (obs.)	
Misinformation prompts, mean ratings (scale 0-7)										
conspiracy theory	5.4	5.5	5.7	0.744		0.091	0.913		57	19
sd	1.9	2.2	1.8							
emotional language [$]	4.9	6.1	5.5	0.018	**	3.748	0.035	**	59	21
sd	1.8	1.3	1.7							
fake experts	5.6	5.9	5.9	0.339		0.373	0.693		50	16
sd	1.9	1.3	1.4							
false dichotomy	5.2	5.6	6.0	0.280		1.455	0.249		59	19
sd	1.8	1.3	1.2							
average misinformation rating	5.3	5.8	5.8	0.058	*	2.367	0.105		75	25
sd	1.6	1.5	1.4							
control	2.1	1.8	2.1	0.276		0.567	0.571		75	25
sd	1.5	1.3	1.6							
Self-efficacy (confidence) prompts mean ratings (scale 1-10)										
identifying accurate information	8.4	8.5	8.8	0.746		1.793	0.500		75	25
sd	2.1	2.2	1.4							
identifying misinformation [$]	7.6	8.3	8.3	0.052	*	2.762	0.073	*	74	25
sd	2.1	1.5	1.5							
talking with friends/family	7.6	8.1	8.4	0.136		2.181	0.124		75	25
sd	1.8	2.0	1.5							
talking with others	6.8	7.6	8.0	0.011	**	7.069	0.002	***	75	25
sd	2.3	1.9	1.7							

* *p*<0.1, ** *p*<0.05, *** *p*<0.01; $ Results for time coefficients on emotional language and identifying misinformation are not robust to Box's conservative epsilon

#### Follow-up interview findings

Of the 11 follow-up interviewees, seven (64%) felt COVID-19 was no longer talked about in their communities, while four (36%) cited specific COVID-19 health misinformation they had continued to encounter. This included two (18%) for masks, one each (9%) for the SARS-CoV-2 virus and vaccines. Seven respondents (64%) indicated that they had used motivational interviewing skills since the training – five (45%) in their personal lives and six (55%) professionally.

Among the seven respondents who reported using motivational interviewing strategies since the training, six (85%) said their experience was positive. One participant shared a specific example of how having an open conversation was a skill translatable to another health-related situation:

“Just recently […] I had a [patient] who had had COVID seriously. Been on a vent, had been⁠—ended up shipping down to Anchorage, and survived it…. We were talking about his survival, his story, his⁠—and whether he needed the pneumonia vaccine and things like that. Some of the things that he needed to think about at this point in his life and how it had had⁠—how the disease had affected him and what he wanted. I loved talking to him even though I think he’s⁠—was resistant to take something else that may affect him negatively. […] I think that it’s the easiest for me is to have that one-on-one conversation and find out a little more where they’re from and asking those open-ended questions and reflecting on what they’re saying before trying to address the solutions.”

When asked about future plans, seven respondents (64%) said they intended to continue using these skills, and four (36%) indicated they planned to share skills with others. When asked about groups to engage in future trainings, seven respondents (64%) suggested specific populations including the public, marginalized groups, health aides, students, first responders, and friends and family. Finally, 10 respondents (91%) indicated that they would want some form of refresher training.

## Discussion

Our findings suggest that both the format and content of the *Building Better Conversations* training was broadly acceptable and modestly improved misinformation recognition. The training also increased participant self-confidence to identify accurate COVID-19 information, identify misinformation, and have constructive conversations around misinformation. Our study responds directly to the growing challenge of health misinformation and disinformation. The training combined evidence-based strategies like building misinformation recognition skills [13–15], motivational interviewing [47, 60, 61], and role plays [50, 51] to encourage participants to identify and address health misinformation and vaccine hesitancy.

This study compliments the growing empirical literature on the effectiveness of motivational interviewing trainings to address vaccine hesitancy. Most existing evidence comes from trainings targeting health care providers. For example, the MOTIVE (MOtivational Interviewing Tool to Increase Vaccine AcceptancE) trial found that a 4-h, in-person educational intervention for pediatric and family practice providers improved the quality of vaccine discussions and reduced refusals for several childhood vaccines [62]. Similar, in-person and online programs for pharmacists and other clinicians have shown that brief motivational interviewing trainings can enhance confidence and communication skills when addressing hesitant patients [63–65].

One recent systematic review of motivational interviewing interventions reported a median training length of nine hours across ten different studies [66]. By contrast, the *Building Better Conversations* training was substantially shorter (two hours) and was designed for a broader, community-based audience that included both health professionals and non-clinical communicators. Despite its brevity and online delivery format, participants demonstrated measurable improvement in self-efficacy and misinformation recognition. This suggests that core motivational interviewing principals and role-play exercises can be effectively used in a 2-h online training. This is a promising avenue for scaling vaccine confidence interventions beyond traditional clinical environments.

Survey and in-depth interview participants identified role plays, interactivity, didactic misinformation content, and motivational interviewing as most helpful in building their confidence to address misinformation. Participants had statistically significant increases in each of four self-efficacy measures we tested from pre- to post-training, in line with findings from prior studies on addressing misinformation through role plays, and interventions that included motivational interviewing [60–63, 67, 68]. For example, a motivational interviewing training employing theatrical role play activities in Portugal found that medical professional participants who frequently interacted with vaccine hesitant patients showed improved knowledge, skills acquisition and self-confidence after two three-hour sessions [68]. Our results show similar participant outcomes but from a shorter online training for a more general audience. Most of our survey respondents and interviewees specifically discussed the benefits of the role plays, suggesting that future trainings incorporating more practical interactive applications of skills may be key to promoting sustained self-efficacy to address misinformation.

Trainees also showed improvements regardless of vaccination status, suggesting a broad translatability of the developed training. However, participants that were fully vaccinated and boosted at baseline were significantly better at a priori misinformation recognition and rated their own self-efficacy about responding to it higher than participants who were not fully vaccinated or boosted prior to training. To our knowledge, these findings are novel, suggesting that exogenous factors may influence both vaccination and capacity to identify misinformation. About a quarter of participants in the training were either unvaccinated or had not received a booster. This suggests that the training may be acceptable for individuals who are vaccine hesitant and aligns with evidence that motivational interviewing and pre-bunking approaches can be delivered to heterogeneous groups [60, 62], including those with lower baseline confidence. This finding also illustrates the widespread nature of vaccine hesitancy, even in public health and medical settings targeted for recruitment. Brief, skills-focused trainings such as this one may be more acceptable and feasible in a mixed-information environment, as the training focused on ways to preserve rapport and incrementally improve misinformation recognition and conversational self-efficacy.

While sample sizes were limited, follow-up findings suggest modest sustainability of improvements attained through the training program. Among three-month follow-up survey participants, 74% attempted conversations around health misinformation using skills learned during the training, and 90% said those conversations were positive and their relationships remained intact or were stronger than before. Current evidence suggests a need for frequent refreshers to sustain motivational interviewing skills in the short term [69, 70], including even light-touch, 15-min refreshers [64]. Repeated measures of misinformation identification and self-efficacy further suggest that these skills were maintained, underscoring the sustainability of even short community-wide trainings like ours. However, these effects may deteriorate over longer time frames.

Though this training was designed and delivered in an Alaska context, the core elements of trusted local messengers/facilitators, misinformation content, motivational interviewing, and role plays of relevant scenarios may be generalizable to other geographic and cultural settings. To adapt this training model elsewhere, implementers should consider a) substituting current, setting-specific vaccination or other health behavior guidance; b) uncovering local misinformation narratives (e.g., around government mandates, fertility, or indigenous/traditional knowledge) and embedding these in the role plays; and c) delivering the training through trusted, place-based facilitators (e.g., public health staff, community health workers, tribal health organizations, or faith/community leaders) who can model non-confrontational conversations. This is consistent with broader guidance on tailoring infodemic management and vaccine confidence interventions to local contexts and messengers [37, 46, 52]. Our audience included both healthcare professionals and non-clinicians and could be adapted to target a different demographic. The online format we used was a practical response to the pandemic, as well as Alaska’s geographic distance and weather. However, the same content could be delivered in hybrid or fully in-person workshops. Several participants requested greater time for the role plays, which could be incorporated into future adaptations.

Several other limitations are worth noting. The most disliked aspect of the training was the shortage of time – specifically for role plays. Indeed, many formal, clinical courses that include motivational interviewing last several hours or even days [40, 41, 62, 64, 66, 69]. While some of the truncated time available for the role plays can be attributed to challenges with facilitator time management, the feasibility of offering longer trainings in our context is limited. Even when scheduling the intended 2-h training, group leaders sometimes asked to shorten sessions to 90 min.

Finally, working in a large, predominantly rural setting like Alaska presents unique challenges. These challenges necessitate flexibility and on-site adaptability to changing environments. For example, Zoom-based trainings depend on reliable internet that was not always present. In one session, a windstorm caused a power outage that prematurely ended the training for some individuals, while sporadic connectivity challenges caused technical issues throughout other trainings. However, remaining flexible and adapting to different contexts than envisioned allowed us to reach a wider audience. For example, the training had been developed assuming each participant would have their own computer with audio/video capacity, but some participants did not have microphones on their computers. In one training, all the participants were in one room with one computer broadcasting the training on a larger screen. These adaptations are to be expected in a real-world setting and were accommodated by the facilitators without real impact on training outcomes or participant perceptions.

## Conclusions

An Alaska-based research team developed and pilot-tested a novel online health misinformation training course that was well-received by a diverse audience of Alaskans. Learners were generally satisfied and particularly enjoyed the opportunity to practice addressing health misinformation in facilitated role plays to learn to *build better conversations.* The findings from this study emphasize the challenges and future opportunities to develop and implement trainings to address the ongoing epidemic of health misinformation. Future trainings could benefit from considering lessons learned, and building upon the strengths identified in this research. Adaptation to local contexts and environments is critical in expanding the reach of misinformation trainings. Much work remains to address the impact of health misinformation, including repairing trust and improving communication.

## Supplementary Information

Supplementary Material 1.

Supplementary Material 2.

Supplementary Material 3.

## Data Availability

The quantitative and qualitative datasets generated and analysed during the current study are not publicly available, as study subjects did not give written consent for their data to be shared publicly. Data are available from the corresponding author Drew Cameron (drew.cameron@yale.edu) upon reasonable request.
