# Supplementary material for: Building better conversations: results of a community-based online health misinformation and motivational interviewing training program in Alaska
Source: BMC Public Health. 2026 Apr 2;26:1542. doi: 10.1186/s12889-026-26611-1 (PMC13169531; doi:10.1186/s12889-026-26611-1)
Supplement: Supplementary file 1 — Supplementary Material 1. [file 12889_2026_26611_MOESM1_ESM.pdf]

Health misinformation in Alaska: *building better conversations*

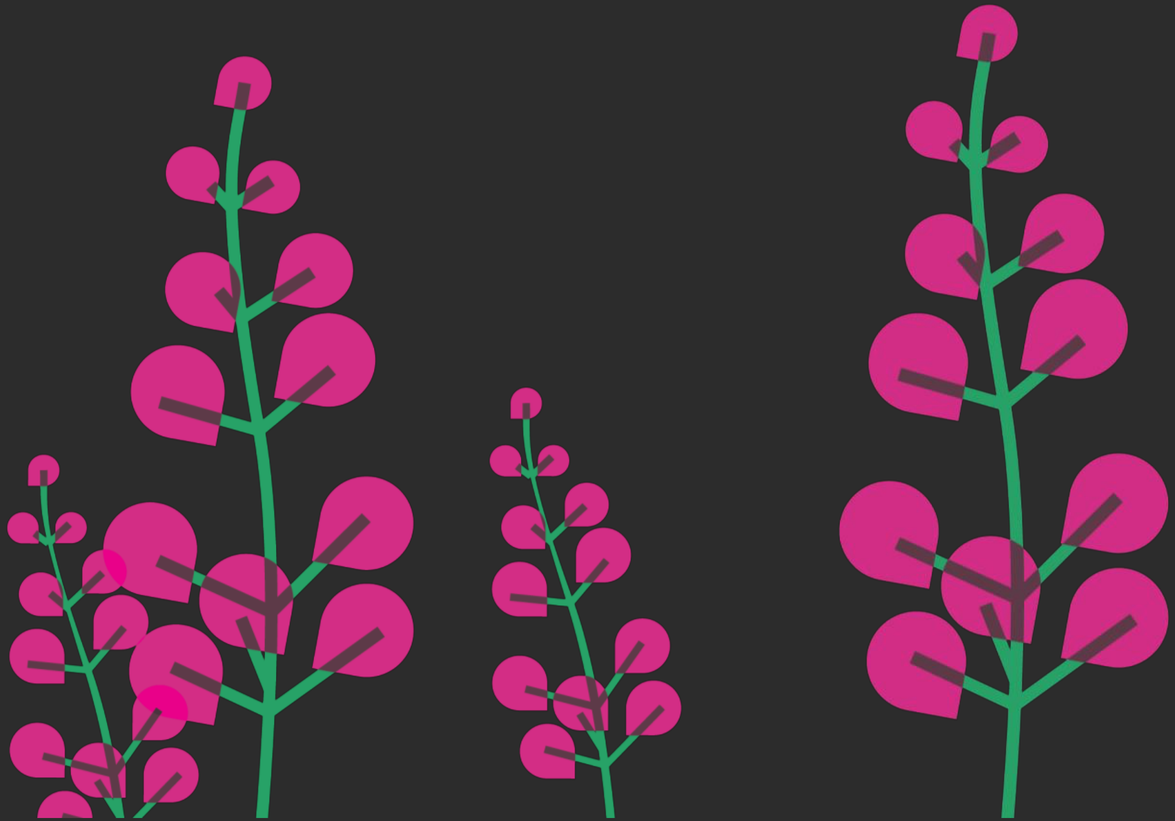

Introductions

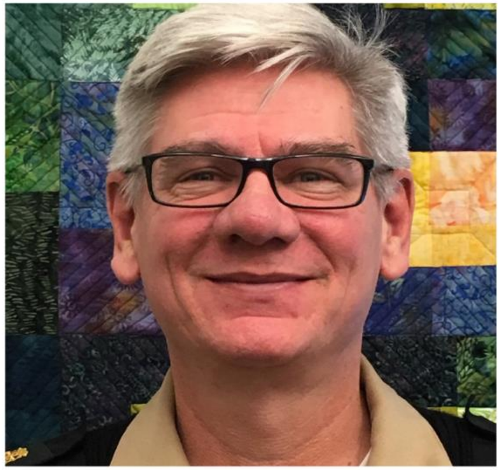

Tom Hennessy

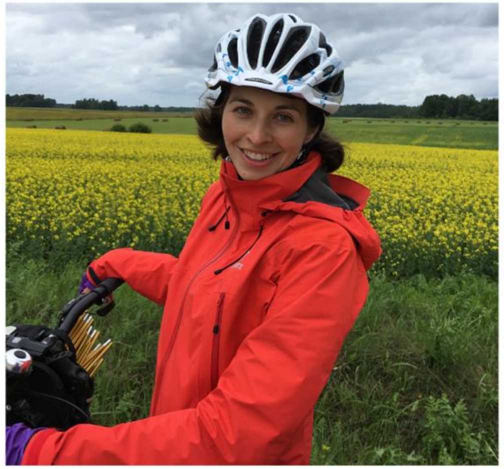

Katie Cueva

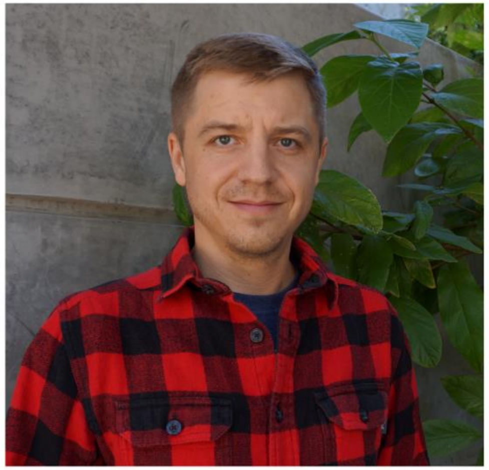

Drew Cameron

ALASKA **HEALTH MISINFORMATION RESPONSE** PROJECT

Research Study

Help us improve this training and bring it to a larger audience!

- Voluntary
- Gift Certificates
- To participate:
  - Electronic Signature – informed consent
  - Pre-survey (5 min)
  - Post-survey (7min)
  - Interviews; focus groups; follow-up survey

Outline

1. Current COVID-19 facts and recommendations

2. Misinformation and disinformation

~ 10 min intermission ~

3. Conversations to address health misinformation

COVID-19
Key Facts and
Current Recommendations

COVID-19 Key Facts

Artist rendering of a Coronavirus

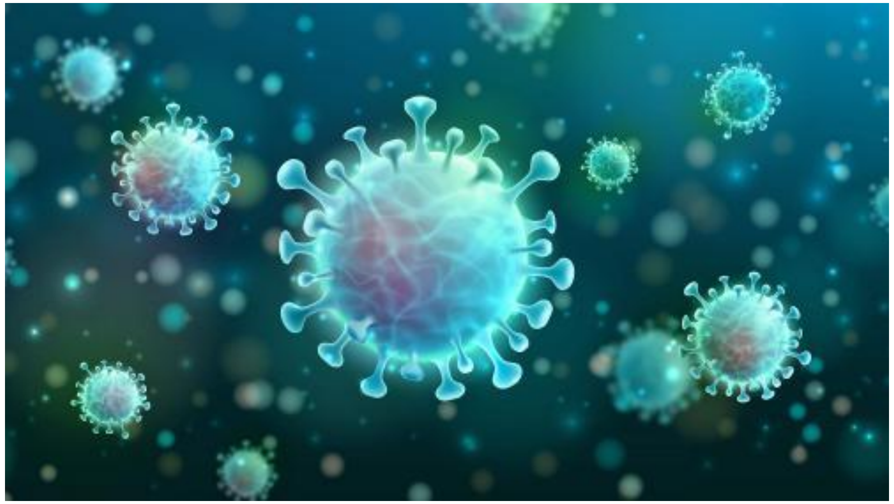

Image (1) from: https://www.fda.gov/emergency-preparedness-and-response/preparedness-research/cellular-signaling-and-immune-correlates-sars-cov-2-infection

COVID-19 is the disease name for an infection

- Caused by new Coronavirus; emerged in China in 2019
- Likely originated in wild bats; only recently infected people

COVID-19 has caused a worldwide outbreak, called a pandemic

- Globally: +500 million infections; +6 million deaths[1]
- US: +80 million infections; +1 million deaths[2]

The pandemic is still going

- June 26: US has 102,000 new infections and 287 deaths each day
- COVID-19 is the third most common cause of death in the US[3]

https://www.cdc.gov/nchs/fastats/leading-causes-of-death.htm

1) CDC COVID Data Tracker, https://covid.cdc.gov/covid-data-tracker/#datatracker-home, Accessed: July 2022.
2) World Health Organization, WHO COVID-19 Dashboard, https://data.who.int/dashboards/covid19/deaths, Accessed: July 2022.
3) CDC, National Center for Health Statistics. https://www.cdc.gov/nchs/fastats/leading-causes-of-death.htm. Accessed: July 2022.

How to Prevent COVID-19[4,5]

1. Get Vaccinated

- Best way to prevent COVID-19
  Safe and effective
- Primary series (one or more) and booster shots
- Recommended for ages 6 months and older
  Some differences in type and doses by age groups

2. Avoid high-risk places

- Especially crowded or poorly ventilated

3. Use Layers of Protection to keep the virus away

- Face masks (esp. N95 respirators) proven to reduce risk
- Social distancing, frequent handwashing, avoid touching eyes, nose and mouth

4. Don't spread COVID-19 to others

- Stay home and arrange to get tested if sick or exposed
- Follow public health recs about how long to stay home and using a mask in public
- Let close contacts know you tested positive

4) CDC COVID-19. https://www.cdc.gov/covid/prevention/index.html, Accessed: July 2022.
5) CDC Respiratory Illnesses. About Physical Distancing and Respiratory Illnesses. https://www.cdc.gov/respiratory-viruses/prevention/physical-distancing.html. Accessed: July 2022

How to Prevent COVID-19[4,5]

1. Get Vaccinated

- Best way to prevent COVID-19
  Safe and effective
- Primary series (one or more) and booster shots
- Recommended for ages 6 months and older
  Some differences in type and doses by age groups

2. Avoid high-risk places

- Especially crowded or poorly ventilated

3. Use Layers of Protection to keep the virus away

- Face masks (esp. N95 respirators) proven to reduce risk
- Social distancing, frequent handwashing, avoid touching eyes, nose and mouth

4. Don't spread COVID-19 to others

- Stay home and arrange to get tested if sick or exposed
- Follow public health recs about how long to stay home and using a mask in public
- Let close contacts know you tested positive

4) CDC COVID-19. https://www.cdc.gov/covid/prevention/index.html, Accessed: July 2022.
5) CDC Respiratory Illnesses. About Physical Distancing and Respiratory Illnesses. https://www.cdc.gov/respiratory-viruses/prevention/physical-distancing.html. Accessed: July 2022

How to Prevent COVID-19[4,5]

1. Get Vaccinated

- Best way to prevent COVID-19
  Safe and effective
- Primary series (one or more) and booster shots
- Recommended for ages 6 months and older
  Some differences in type and doses by age groups

2. Avoid high-risk places

- Especially crowded or poorly ventilated

3. Use Layers of Protection to keep the virus away

- Face masks (esp. N95 respirators) proven to reduce risk
- Social distancing, frequent handwashing, avoid touching eyes, nose and mouth

4. Don't spread COVID-19 to others

- Stay home and arrange to get tested if sick or exposed
- Follow public health recs about how long to stay home and using a mask in public
- Let close contacts know you tested positive

4) CDC COVID-19. https://www.cdc.gov/covid/prevention/index.html, Accessed: July 2022.
5) CDC Respiratory Illnesses. About Physical Distancing and Respiratory Illnesses. https://www.cdc.gov/respiratory-viruses/prevention/physical-distancing.html. Accessed: July 2022

How to Prevent COVID-19[4,5]

1. Get Vaccinated

- Best way to prevent COVID-19
  Safe and effective
- Primary series (one or more) and booster shots
- Recommended for ages 6 months and older
  Some differences in type and doses by age groups

2. Avoid high-risk places

- Especially crowded or poorly ventilated

3. Use Layers of Protection to keep the virus away

- Face masks (esp. N95 respirators) proven to reduce risk
- Social distancing, frequent handwashing, avoid touching eyes, nose and mouth

4. Don't spread COVID-19 to others

- Stay home and arrange to get tested if sick or exposed
- Follow public health recs about how long to stay home and using a mask in public
- Let close contacts know you tested positive

4) CDC COVID-19. https://www.cdc.gov/covid/prevention/index.html, Accessed: July 2022.
5) CDC Respiratory Illnesses. About Physical Distancing and Respiratory Illnesses. https://www.cdc.gov/respiratory-viruses/prevention/physical-distancing.html. Accessed: July 2022

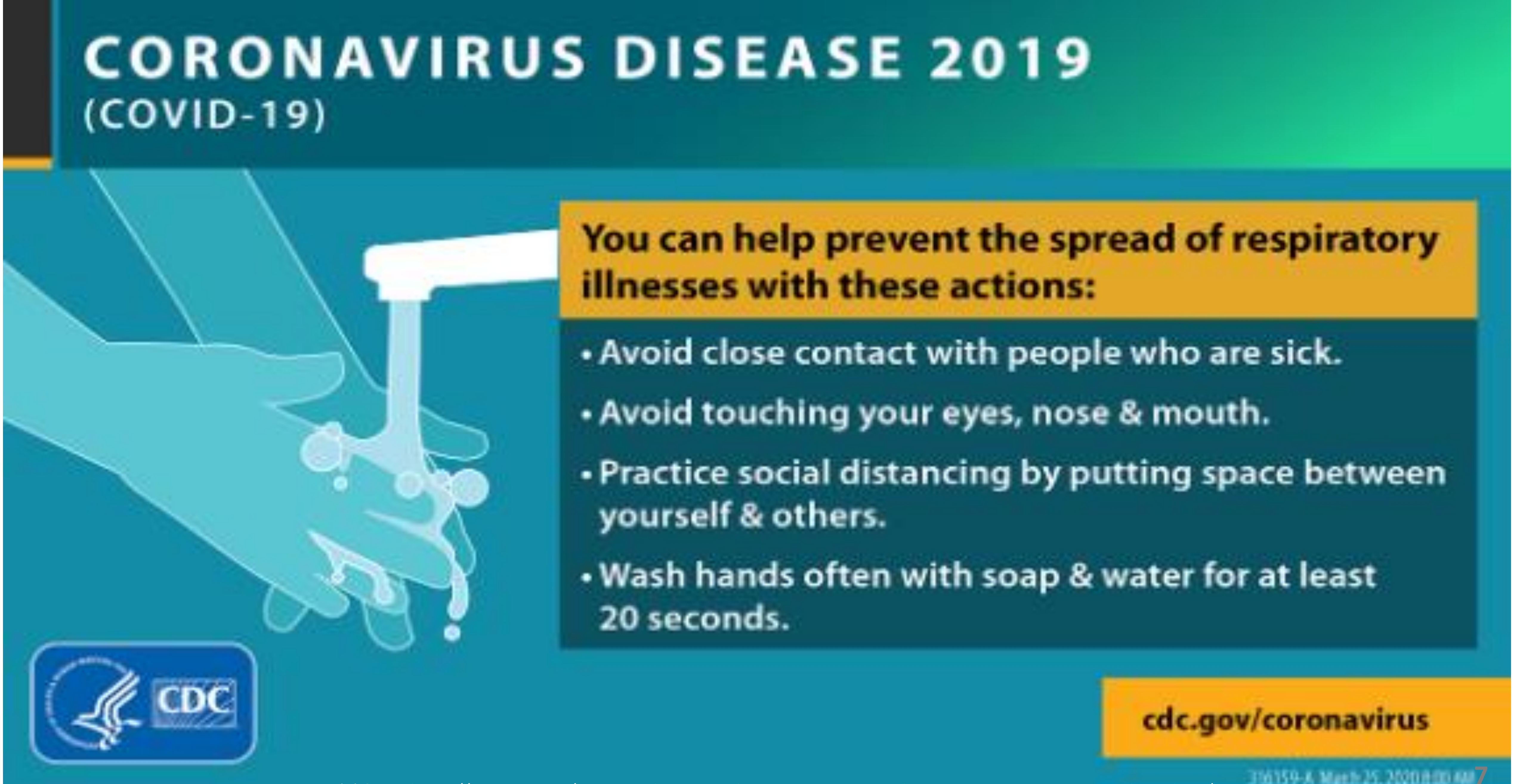

Image (2) from: https://nutrition.org/nutritional-epidemiologists-encounter-with-coronavirus-covid-19-in-wuhan-china/

COVID-19 prevention – We are not powerless

Use layered Prevention

- Vaccines
- Masks
- Testing
- Physical distancing
- Ventilation

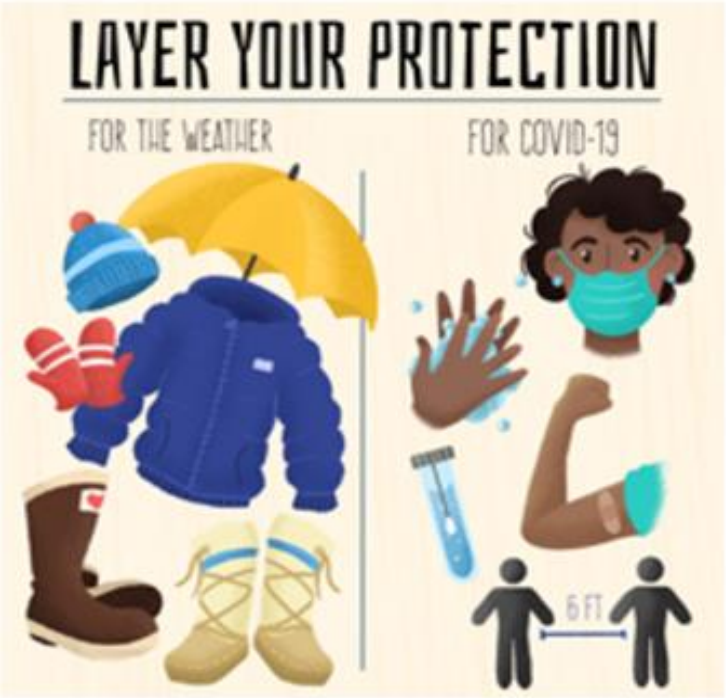

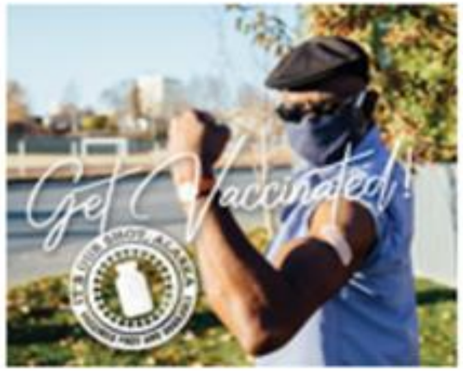

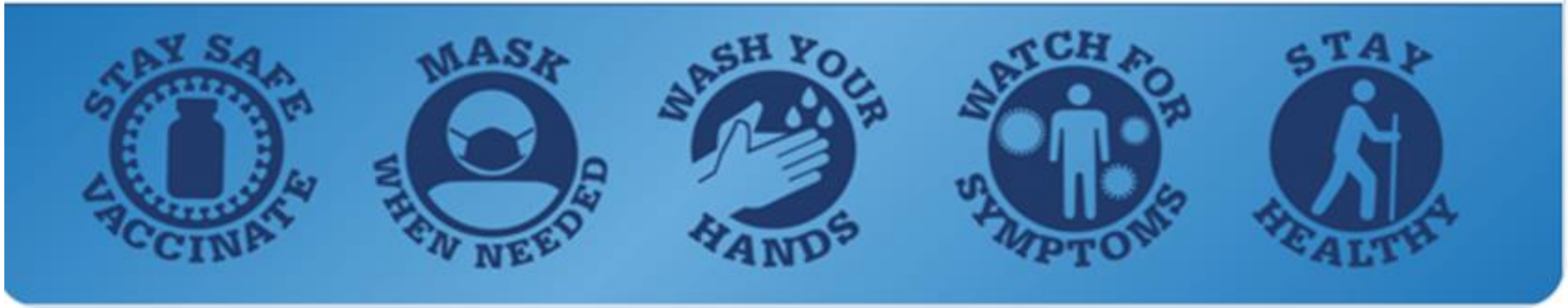

Image (3) from: Alaska Department of Health, July 2022

Treating COVID-19

Many COVID-19 infections are mild and go away without medications

- The average length of illness is one to two weeks

Seek medical care if you have warning signs

- Shortness of breath, chest pain, extreme fatigue, confusion, blue lips or skin

Early medical treatment can save lives

- Approved anti-viral or antibody treatments can be lifesaving for very ill people or those with health problems

Don’t take unproven treatments

- The internet is full of bad advice; unproven treatments with vitamins, antibiotics, and anti-parasite drugs
- Get medical care from a licensed healthcare professional
- The National Institutes of Health guidance for treatment is the best advice for treating COVID-19 https://www.covid19treatmentguidelines.nih.gov

Key Takeaways

COVID-19 is a new and very serious infection

- As we learn more, advice on treatment and prevention will change
- This is normal - it takes time for medical science to find answers

Getting vaccinated is your best defense against COVID-19

- Vaccines are the safest and best way to build your immunity to COVID-19 https://www.vaccines.gov/search/

If you are sick or exposed to someone with COVID-19

- Stay home, arrange for testing
- Seek professional medical care if you have warning signs https://www.cdc.gov/coronavirus/2019-ncov/you-health/quarantine-isolation.html

Misinformation and Disinformation

Misinformation and Disinformation[6]

Misinformation is false information that is spread either by mistake or with the intent to mislead.

When there's intent to mislead, we call this disinformation.

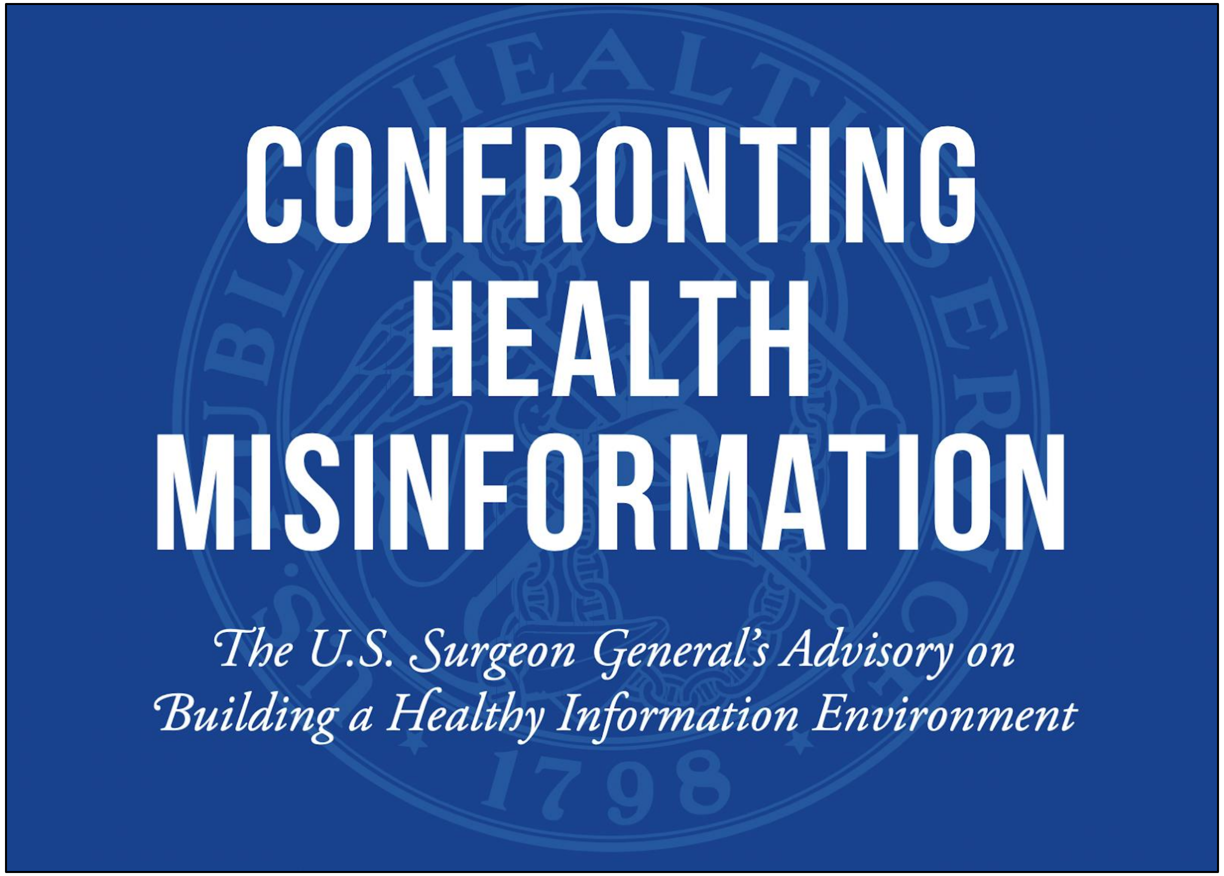

Image (4) from: . https://www.hhs.gov/surgeongeneral/reports-and-publications/health-misinformation/index.html

6) U.S. Department of Health and Human Services. Health Misinformation. https://www.hhs.gov/surgeongeneral/reports-and-publications/health-misinformation/index.html. Accessed: July 2022.

Common misinformation tactics[7]

Tactic	Explanation
Emotional Language	
Incoherence	
False choices	
Scapegoating	
Personal Attacks	
Fake experts	
Setting impossible expectations	
Conspiracies	

7) Inoculation Science. https://inoculation.science/. Accessed: July 2022

Common misinformation tactics[7]

Tactic	Explanation
**Emotional Language**	Language that encourages negative emotions like fear, outrage (makes posts go viral online)
Incoherence	
False choices	
Scapegoating	
Personal Attacks	
Fake experts	
Setting impossible expectations	
Conspiracies	

7) Inoculation Science. https://inoculation.science/. Accessed: July 2022

Common misinformation tactics[7]

Tactic	Explanation
**Emotional Language**	Language that encourages negative emotions like fear, outrage (makes posts go viral online)
Incoherence	
False choices	
Scapegoating	
Personal Attacks	
Fake experts	
Setting impossible expectations	
Conspiracies	

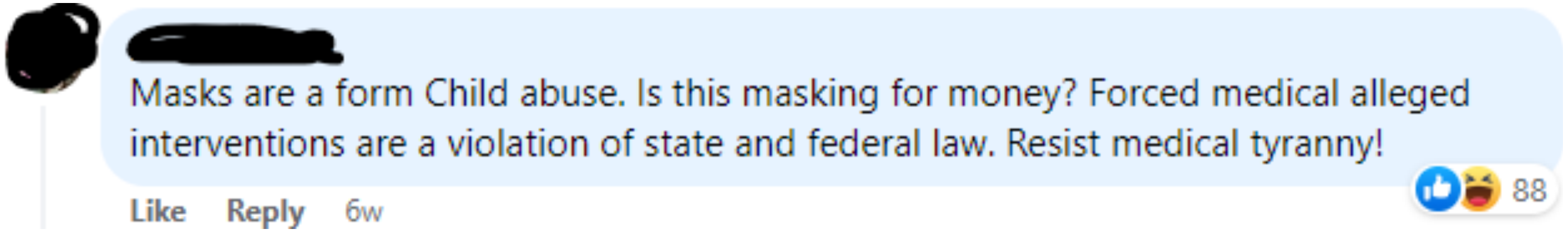

7) Inoculation Science. https://inoculation.science/. Accessed: July 2022

Common misinformation tactics[7]

Tactic	Explanation
**Emotional Language**	Language that encourages negative emotions like fear, outrage (makes posts go viral online)
Incoherence	
False choices	
Scapegoating	
Personal Attacks	
Fake experts	
Setting impossible expectations	
Conspiracies	

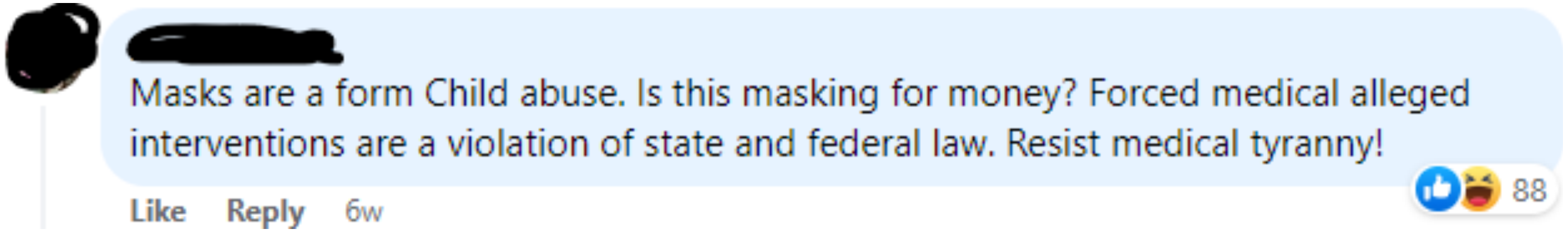

https://inoculation.science/inoculation-videos/emotional-language/

7) Inoculation Science. https://inoculation.science/. Accessed: July 2022

Common misinformation tactics[7]

Tactic	Explanation
Emotional Language	
**Incoherence**	Illogical reasoning; using two or more arguments that cannot be true at the same time to prove a point
False choices	
Scapegoating	
Personal Attacks	
Fake experts	
Setting impossible expectations	
Conspiracies	

7) Inoculation Science. https://inoculation.science/. Accessed: July 2022

Common misinformation tactics[7]

Tactic	Explanation
Emotional Language	
**Incoherence**	Illogical reasoning; using two or more arguments that cannot be true at the same time to prove a point
False choices	
Scapegoating	
Personal Attacks	https://inoculation.science/inoculation-videos/incoherence/
Fake experts	
Setting impossible expectations	
Conspiracies	

7) Inoculation Science. https://inoculation.science/. Accessed: July 2022

Common misinformation tactics[7]

Tactic	Explanation
Emotional Language	
Incoherence	
**False choices**	Using a limited number of choices/sides to an issue when more options are available
Scapegoating	
Personal Attacks	
Fake experts	
Setting impossible expectations	
Conspiracies	

7) Inoculation Science. https://inoculation.science/. Accessed: July 2022

Common misinformation tactics[7]

Tactic	Explanation
Emotional Language	
Incoherence	
**False choices**	Using a limited number of choices/sides to an issue when more options are available
Scapegoating	
Personal Attacks	
Fake experts	
Setting impossible expectations	
Conspiracies	

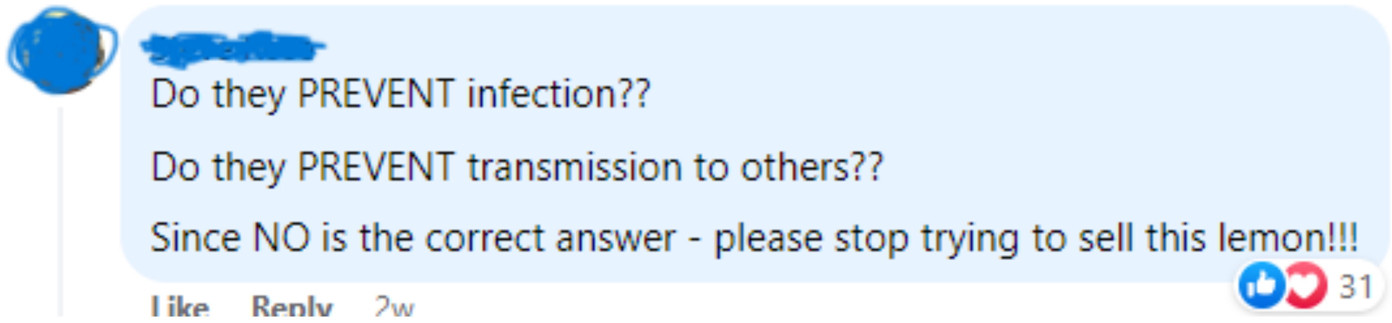

7) Inoculation Science. https://inoculation.science/. Accessed: July 2022

Common misinformation tactics[7]

Tactic	Explanation
Emotional Language	
Incoherence	
**False choices**	Using a limited number of choices/sides to an issue when more options are available
Scapegoating	
Personal Attacks	
Fake experts	
Setting impossible expectations	
Conspiracies	

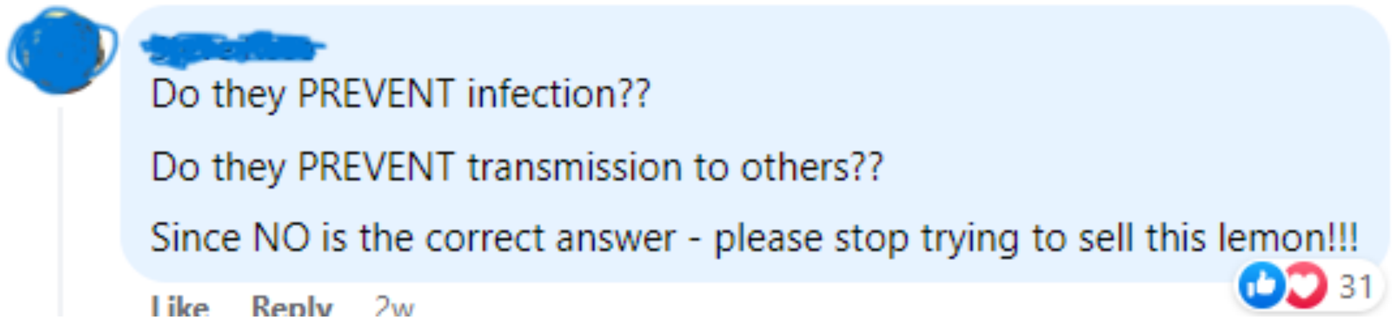

https://inoculation.science/inoculation-videos/false-dichotomy/

7) Inoculation Science. https://inoculation.science/. Accessed: July 2022

Common misinformation tactics[7]

Tactic	Explanation
Emotional Language	
Incoherence	
False choices	
**Scapegoating**	Singling out a person or group for a particular problem
Personal Attacks	
Fake experts	
Setting impossible expectations	
Conspiracies	

7) Inoculation Science. https://inoculation.science/. Accessed: July 2022

Common misinformation tactics[7]

Tactic	Explanation
Emotional Language	
Incoherence	
False choices	
**Scapegoating**	Singling out a person or group for a particular problem
Personal Attacks	
Fake experts	
Setting impossible expectations	
Conspiracies	

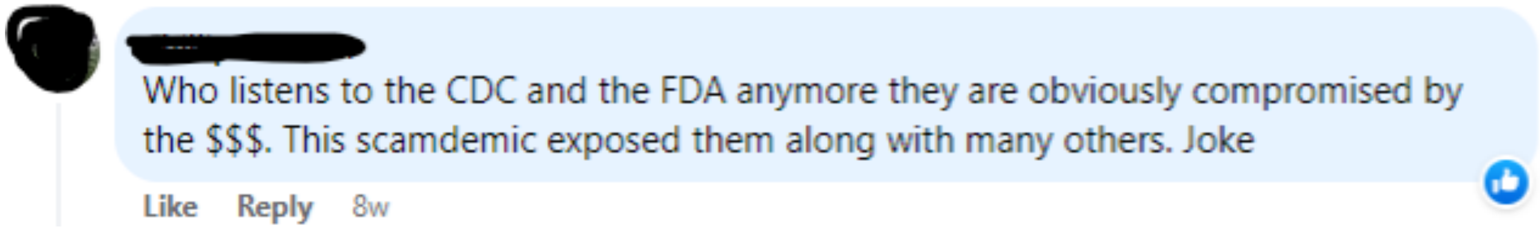

7) Inoculation Science. https://inoculation.science/. Accessed: July 2022

Common misinformation tactics[7]

Tactic	Explanation
Emotional Language	
Incoherence	
False choices	
**Scapegoating**	Singling out a person or group for a particular problem
Personal Attacks	
Fake experts	
Setting impossible expectations	
Conspiracies	

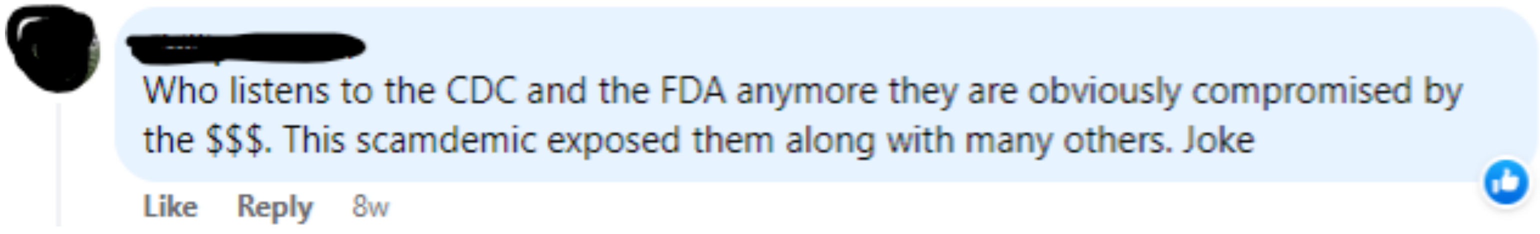

https://inoculation.science/inoculation-videos/scapegoating/

7) Inoculation Science. https://inoculation.science/. Accessed: July 2022

Common misinformation tactics[7]

Tactic	Explanation
Emotional Language	
Incoherence	
False choices	
Scapegoating	
**Personal Attacks**	When someone attacks the person making the argument instead of the argument itself (a.k.a., "ad hominem attacks")
Fake experts	
Setting impossible expectations	
Conspiracies	

7) Inoculation Science. https://inoculation.science/. Accessed: July 2022

Common misinformation tactics[7]

Tactic	Explanation
Emotional Language	
Incoherence	
False choices	
Scapegoating	
**Personal Attacks**	When someone attacks the person making the argument instead of the argument itself (a.k.a., “ad hominem attacks”)

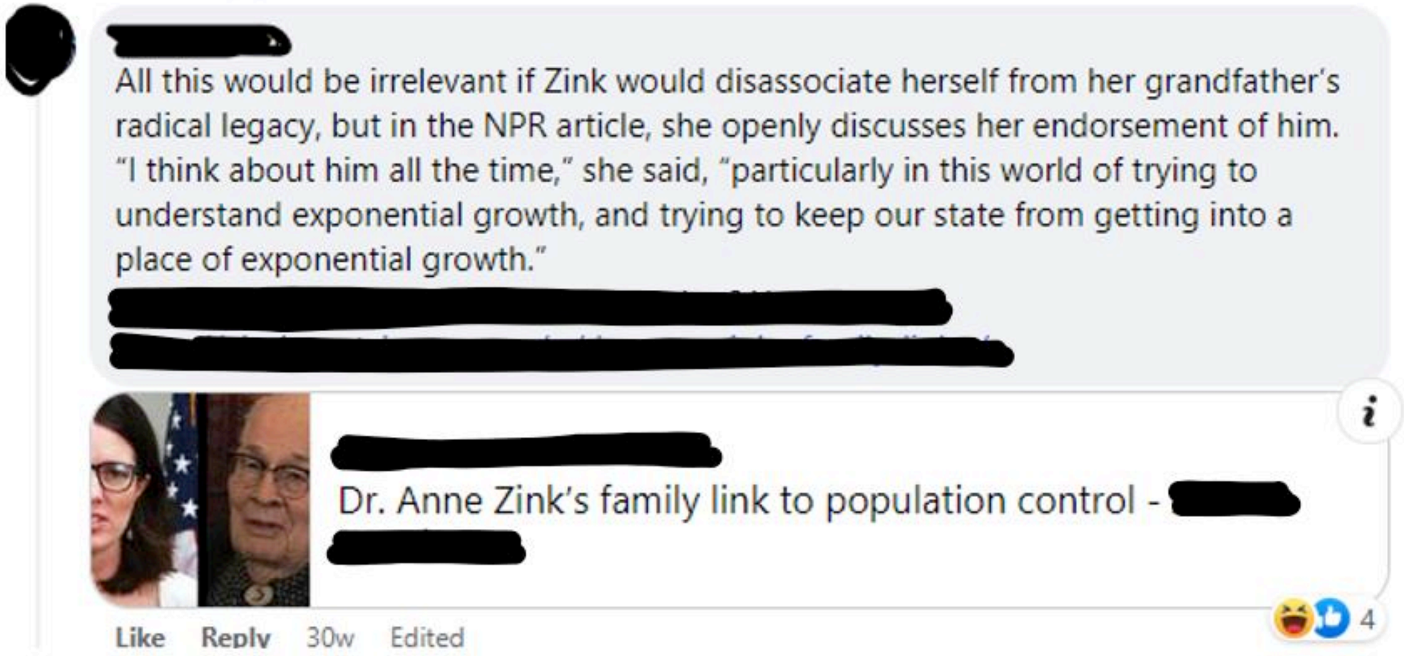

7) Inoculation Science. https://inoculation.science/. Accessed: July 2022

Common misinformation tactics[7]

Tactic	Explanation
Emotional Language	
Incoherence	
False choices	
Scapegoating	
**Personal Attacks**	When someone attacks the person making the argument instead of the argument itself (a.k.a., “ad hominem attacks”)

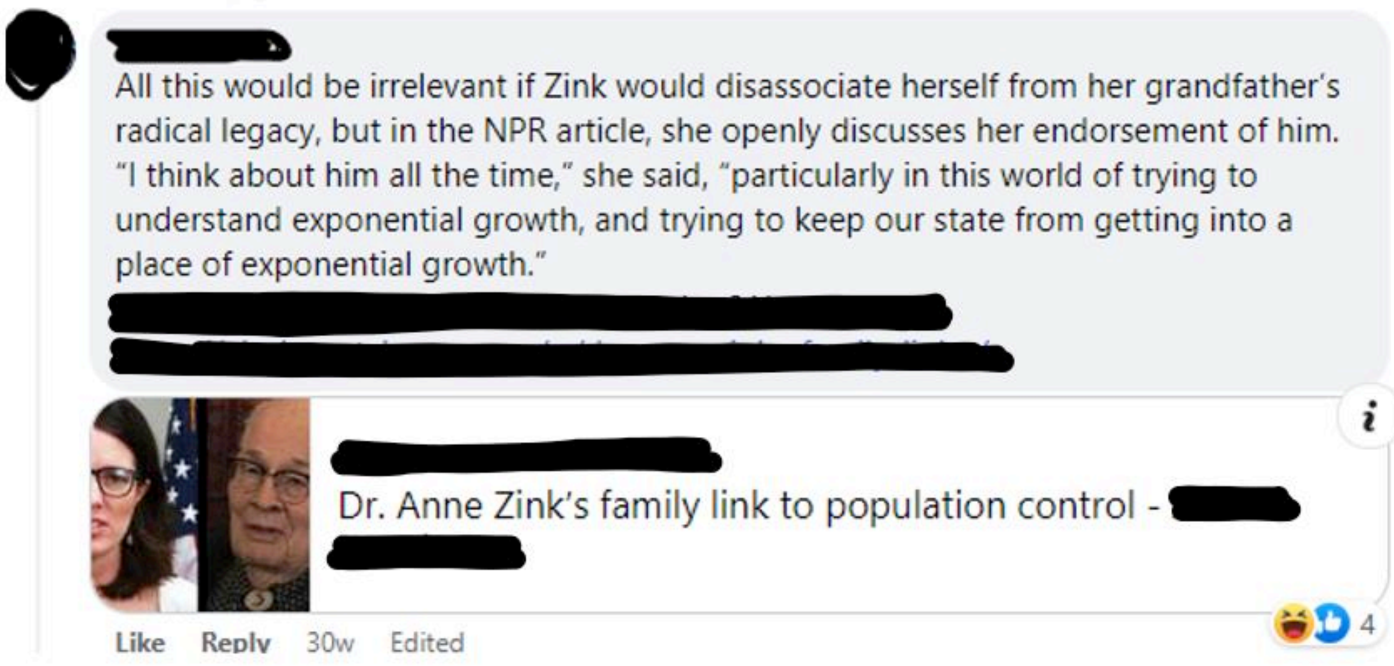

https://inoculation.science/inoculation-videos/ad-hominem-attack/

7) Inoculation Science. https://inoculation.science/. Accessed: July 2022

Common misinformation tactics[7]

Tactic	Explanation
Emotional Language	
Incoherence	
False choices	
Scapegoating	
Personal Attacks	
**Fake experts**	Presenting unqualified individuals/institutions as sources of credible information
Setting impossible expectations	
Conspiracies	

7) Inoculation Science. https://inoculation.science/. Accessed: July 2022

Common misinformation tactics[7]

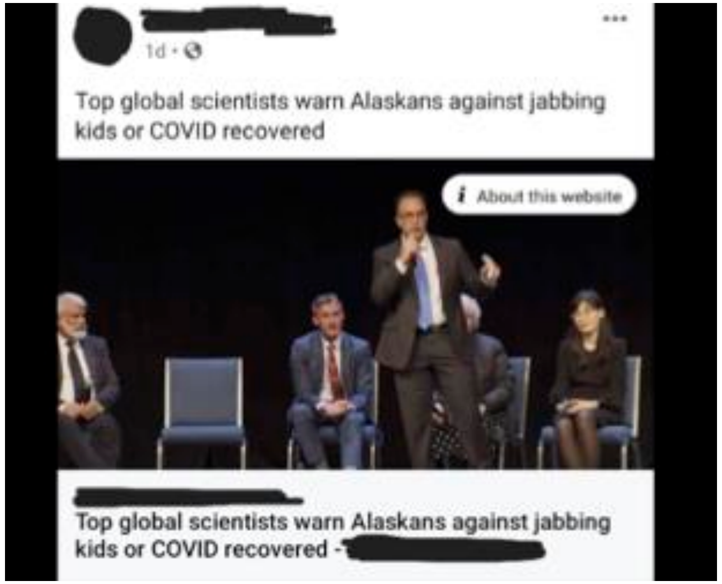

Tactic	Explanation
Emotional Language	
Incoherence	
False choices	
Scapegoating	
Personal Attacks	
**Fake experts**	Presenting unqualified individuals/institutions as sources of credible information
Setting impossible expectations	
Conspiracies	

7) Inoculation Science. https://inoculation.science/. Accessed: July 2022

Common misinformation tactics[7]

Tactic	Explanation
Emotional Language	
Incoherence	
False choices	
Scapegoating	
Personal Attacks	
Fake experts	
**Setting impossible expectations**	Demanding unrealistic standards of certainty before acting on science
Conspiracies	

7) Inoculation Science. https://inoculation.science/. Accessed: July 2022

Common misinformation tactics[7]

Tactic	Explanation
Emotional Language	
Incoherence	
False choices	
Scapegoating	
Personal Attacks	
Fake experts	
**Setting impossible expectations**	Demanding unrealistic standards of certainty before acting on science
Conspiracies	

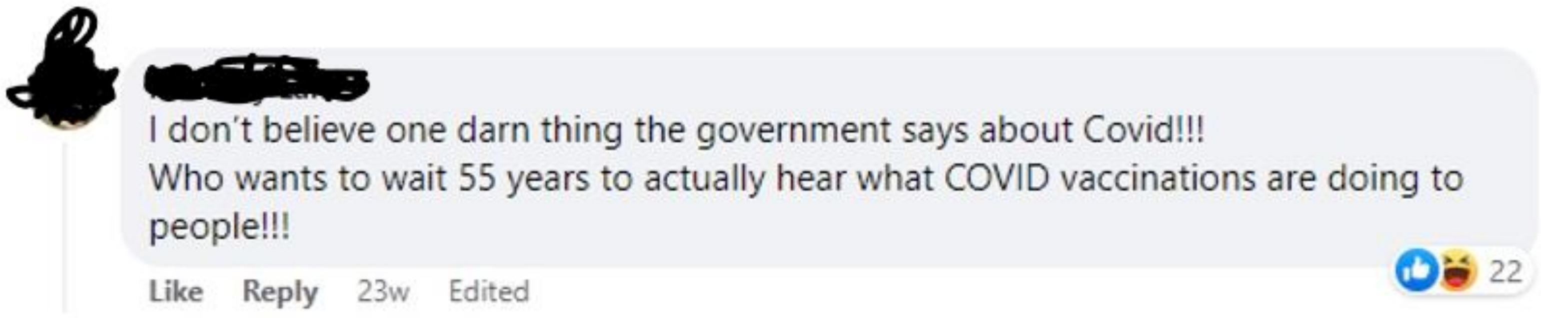

7) Inoculation Science. https://inoculation.science/. Accessed: July 2022

Common misinformation tactics[7]

Tactic	Explanation
Emotional Language	
Incoherence	
False choices	
Scapegoating	
Personal Attacks	
Fake experts	
Setting impossible expectations	
**Conspiracies**	Proposing that a secret plan exists / Nefarious scheme to hide the truth

7) Inoculation Science. https://inoculation.science/. Accessed: July 2022

Common misinformation tactics[7]

Tactic	Explanation
Emotional Language	
Incoherence	
False choices	
Scapegoating	
Personal Attacks	
Fake experts	
Setting impossible expectations	
**Conspiracies**	Proposing that a secret plan exists / Nefarious scheme to hide the truth

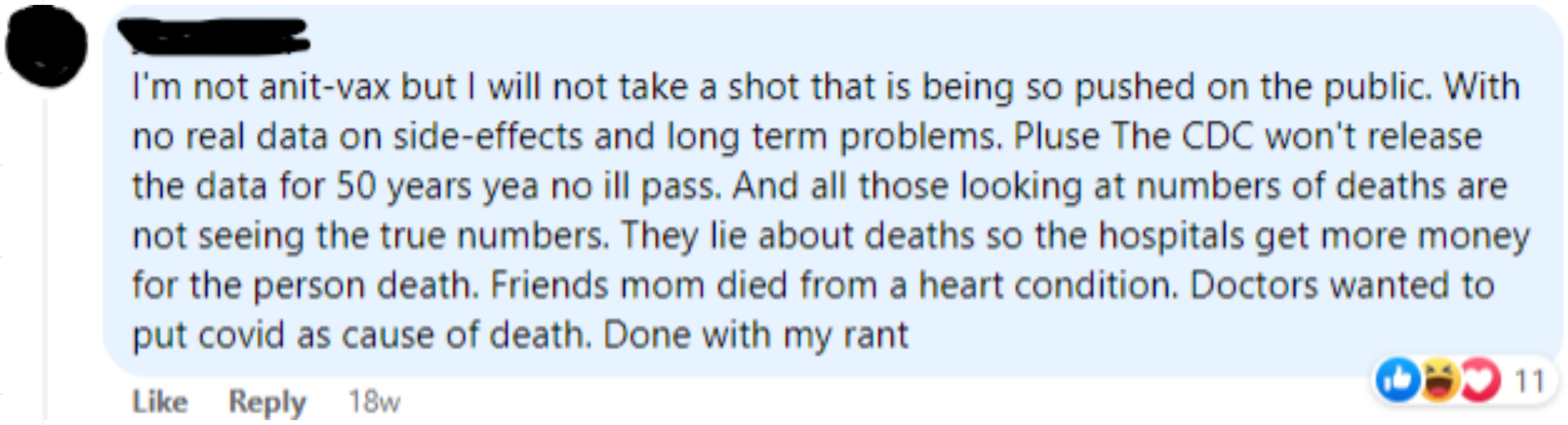

7) Inoculation Science. https://inoculation.science/. Accessed: July 2022

Intermission

- 10-minute bio break
- Play “go viral”: https://inoculation.science/inoculation-games/go-viral/

Skills to Address Misinformation

1. Listen[8]

- The best way to change someone's mind about misinformation is to listen to their fears and why they believe what they do.
- Try not to focus on the content or false claim; instead, focus on the wider issue and how they feel about that issue
- While sometimes it can be tempting to "fact check" and prove someone wrong, this approach can often shut down a conversation

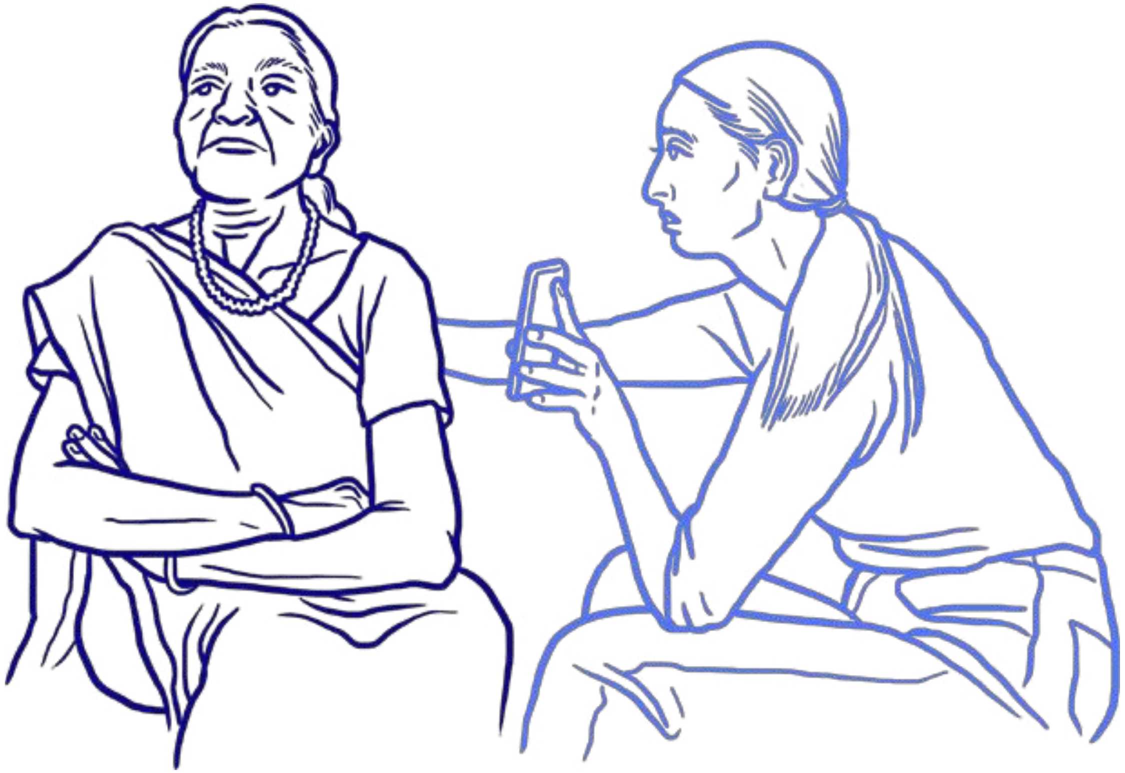

Image (5) from: https://www.hhs.gov/sites/default/files/health-misinformation-toolkit-english.pdf

8) Surgeon General's Health Misinformation Toolkit. https://www.hhs.gov/sites/default/files/health-misinformation-toolkit-english.pdf, Accessed: July 2022

2. Empathize[8]

- When talking to a friend or family member, emphasize the fact that you understand that there are often reasons why people find it difficult to trust certain sources of information.
- Ask questions to learn where they're coming from.
- Admit that you have struggled and continue to struggle with knowing what is true and false.
- Where possible, talk about times when you have fallen for misinformation, and explain why you were susceptible.

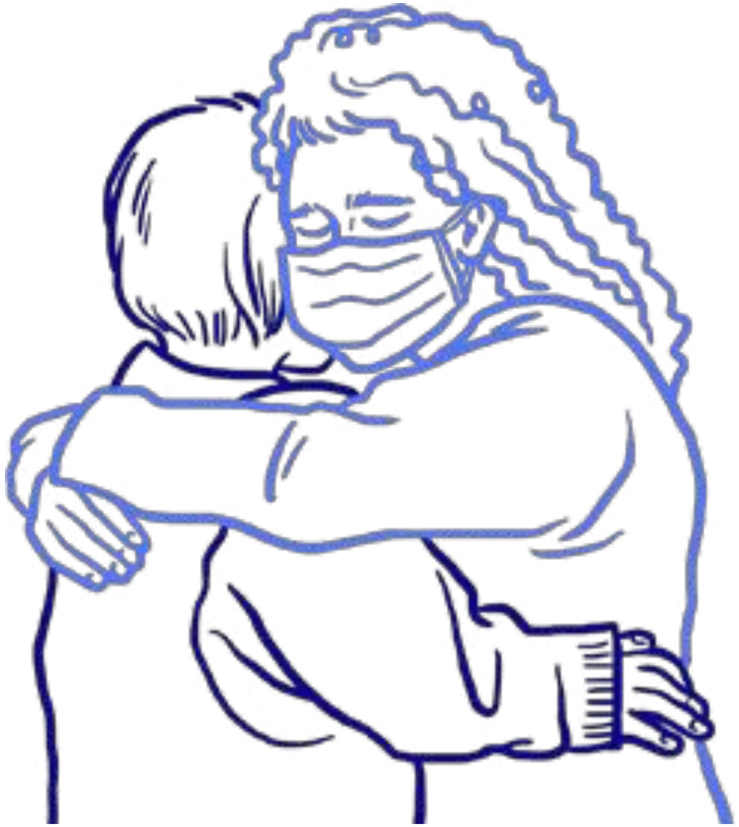

Image (5) from: https://www.hhs.gov/sites/default/files/health-misinformation-toolkit-english.pdf

8) Surgeon General's Health Misinformation Toolkit. https://www.hhs.gov/sites/default/files/health-misinformation-toolkit-english.pdf, Accessed: July 2022

3. Point to credible sources[8]

- Underscore that finding accurate information can be hard, especially during events like the pandemic when the information is constantly changing (which will always happen with a new virus or disease)

- Emphasize the need to find credible sources, who are not positioned to personally profit or to gain power or influence

- Remind them that an expert on one topic might not be an expert to turn to on another topic

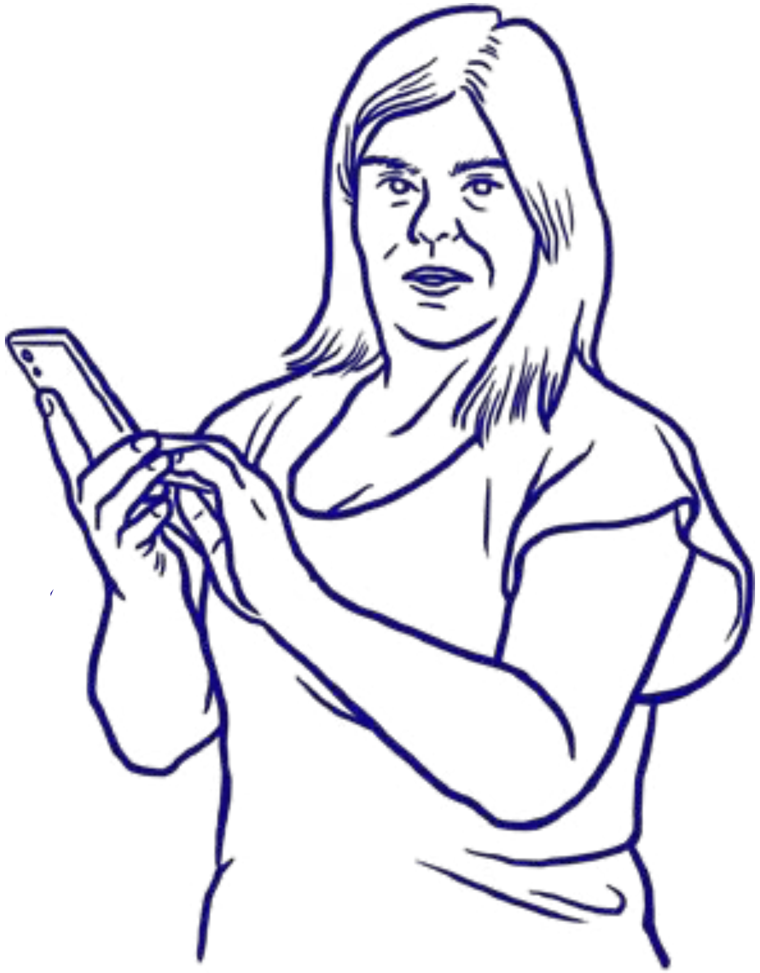

Image (5) from: https://www.hhs.gov/sites/default/files/health-misinformation-toolkit-english.pdf

8) Surgeon General's Health Misinformation Toolkit. https://www.hhs.gov/sites/default/files/health-misinformation-toolkit-english.pdf, Accessed: July 2022

4. Don't publicly shame[8]

- Where possible, try to have conversations one-on-one, either face to face or via direct messages on social media sites. Remember, no one likes to appear wrong

- Having conversations in the comments under a post has the potential to backfire or means more people might see the misinformation

- A caring tone of voice could help more people. Be gentle in your replies and remember to listen and be empathetic

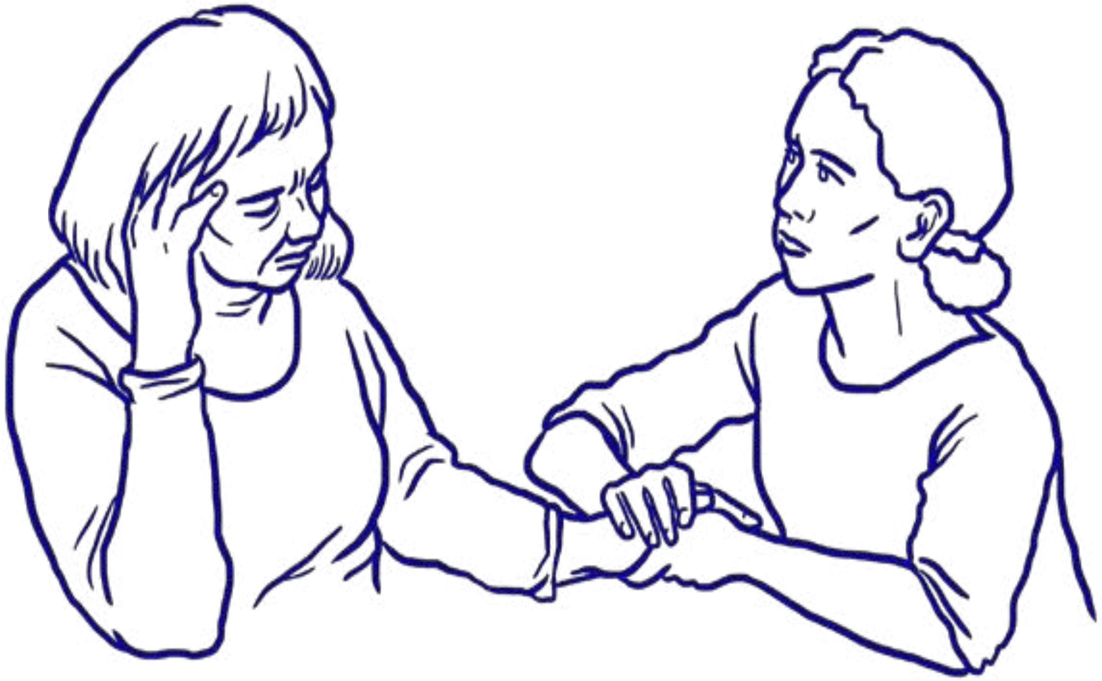

Image (5) from: https://www.hhs.gov/sites/default/files/health-misinformation-toolkit-english.pdf

8) Surgeon General's Health Misinformation Toolkit. https://www.hhs.gov/sites/default/files/health-misinformation-toolkit-english.pdf, Accessed: July 2022

5. Use inclusive language[8]

- Where possible, use language that makes it clear that you see yourself being impacted in the same way.
- Show that you sometimes struggle to figure out whom or what to trust.
- Be willing to share an example of when you struggled to figure out whether something was true

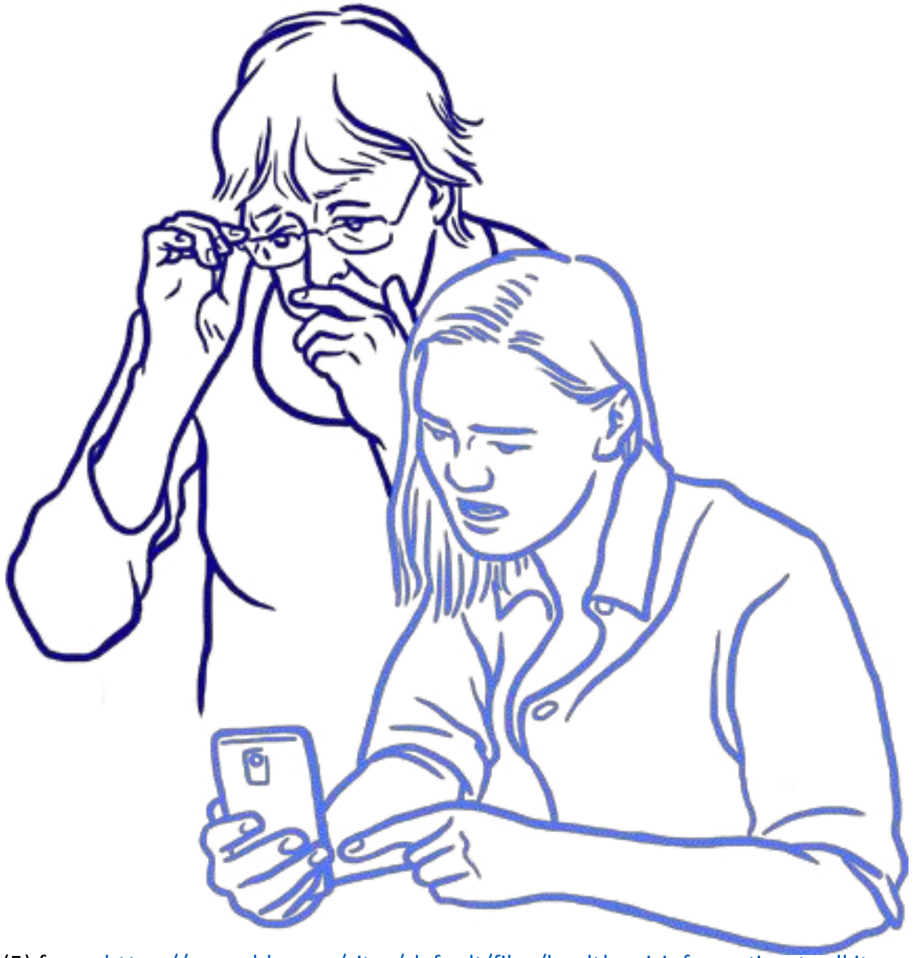

Image (5) from: https://www.hhs.gov/sites/default/files/health-misinformation-toolkit-english.pdf

8) Surgeon General's Health Misinformation Toolkit. https://www.hhs.gov/sites/default/files/health-misinformation-toolkit-english.pdf, Accessed: July 2022

Motivational Interviewing

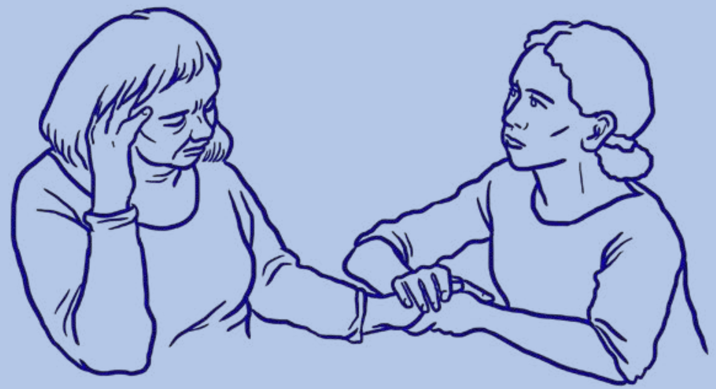

Image (5) from: https://www.hhs.gov/sites/default/files/health-misinformation-toolkit-english.pdf

- *Motivational interviewing is an empathic, person-centered approach that prepares people for change by helping them resolve uncertainty, enhance internal motivation, and build confidence to change.*

O = open ended questions

A = affirmations

R = reflexive listening

S = summarize the conversation

Image from: https://www.hhs.gov/sites/default/files/health-misinformation-toolkit-english.pdf

OARS - Motivational Interviewing

Open ended questions

- Invite the person to tell their story or voice their concerns in their own words
- Don’t lead them in a specific direction.
- Be willing to listen to the WHOLE response
- Avoid “yes” or “no” questions.

Help me understand…

Can you tell me your concerns about …

How would you like things to be different?

What do you want to do next?

OARS - Motivational Interviewing

Affirmations

- Recognize the person's strengths
- Acknowledge behaviors that lead in a direction of positive change, no matter how large or small.
- Build confidence in a genuine and consistent way.

I can see you really care about the health and safety of your family

Its clear that you've taken time to inform yourself

You are clearly very resourceful

I've really enjoyed talking to you about this

OARS - Motivational Interviewing

Reflective Listening

This is the hardest part to do well!

- Repeat or rephrase what the person has said
- Or, reflect on the feelings that the person's explanation evokes
- Your voice turns DOWN at the end of the sentence, rather than asking a question

So, you feel concerned about …

It sounds like you want to know more about vaccine safety …

You're wondering if …

You've been wrestling with some complicated issues

OARS - Motivational Interviewing

Summarize the conversation

- Begin with a statement that you are making a summary of the conversation
- Give special attention to CHANGE STATEMENTS
- If the person expresses uncertainty, include both sides in the summary statement
- Include concise objective information and
- End with an invitation

Let me see if I understand so far

You're worried about vaccine safety, but you also don't want your kids to get sick from the virus

I've been paying close attention to the findings on vaccine trials. Can I share some of what I've learned with you?

Did I miss anything? Do you have other concerns?

MI Example

M.I. Practice 1: Family reunion

- **A. MI Practice**: Your friend is telling you about a family reunion they have been invited to, but they are concerned that the hostess has asked that everyone (including kids) are vaccinated. Your friend's kids are not vaccinated.

- **B. Misinformed**: You're looking forward to the reunion, but you've heard that giving kids the COVID vaccine could impact their fertility.

B. I'm really excited to go to my family reunion - we're having caribou! But they want my kids vaccinated against COVID-19 … and I have concerns…

A. Practice your MI skills

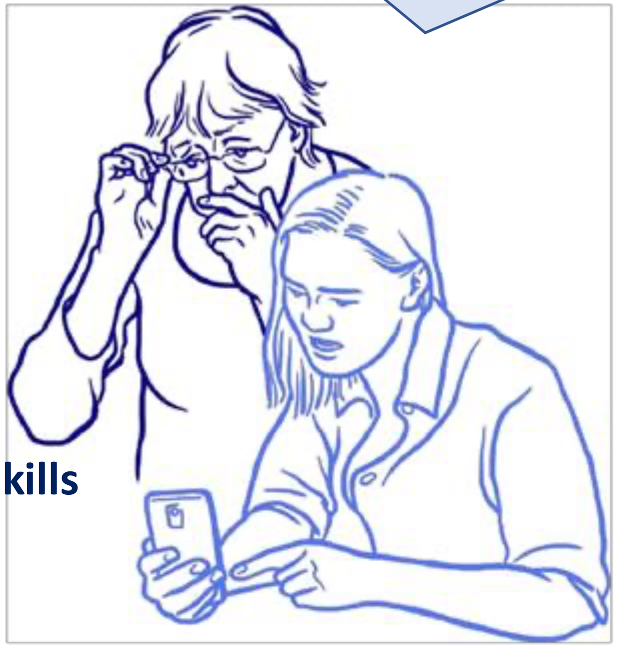

B. Misinformed

<u>Remember</u>

O – Open ended questions
A – Affirmations
R – Reflective listening
S – Summaries

(Person A = Alphabetical by first name)

Image (5) from: https://www.hhs.gov/sites/default/files/health-misinformation-toolkit-english.pdf

M.I. Practice 1: Family reunion

SWITCH!

- **B. MI Practice**: Your friend is telling you about a family reunion they have been invited to, but they are concerned that the hostess has asked that everyone (including kids) are vaccinated. Your friend's kids are not vaccinated.

- **A. Misinformed**: You're looking forward to the reunion, but you've heard that giving kids the COVID vaccine could impact their fertility.

A. I'm really excited to go to my family reunion - we're having caribou! But they want my kids vaccinated against COVID-19 … and I have concerns…

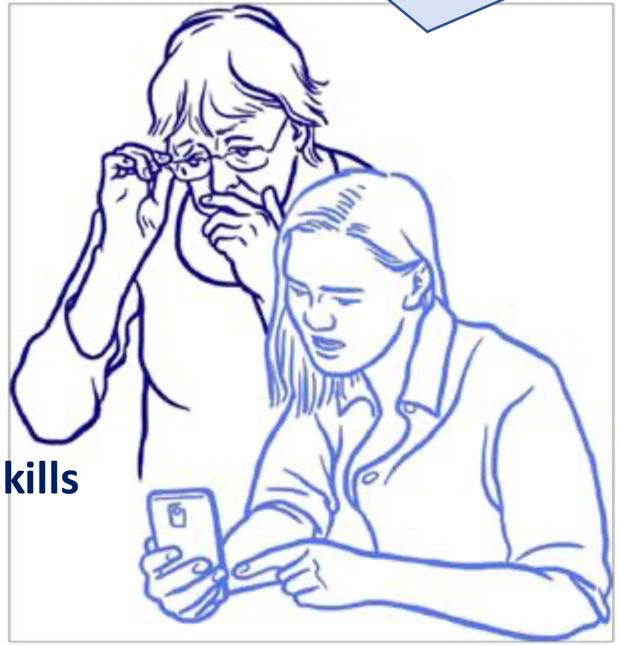

B. Practice your MI skills

A. Misinformed

<u>Remember</u>
O – Open ended questions
A – Affirmations
R – Reflective listening
S – Summaries

(Person A = Alphabetical by first name)

Image (5) from: https://www.hhs.gov/sites/default/files/health-misinformation-toolkit-english.pdf

Debrief

- What worked well?
- What didn't work?
- Was there resistance?
- How was the resistance handled?

Countering resistance

Signs of resistance

- Interrupting
  - Talking over, cutting off
- Arguing
  - Challenging, discounting, hostility
- Ignoring

  Inattention, non-response, sidetracking
- Denying

  Blaming, Disagreeing, minimizing, reluctance

How to counter

- Reflection

  Acknowledge disagreement or emotion
- Emphasize personal choice & control

  Assure them THEY determine what happens
- Clarification

  Verify that you understand their perspective
- Shifting focus

  Talk about points of agreement

M.I. Practice 2: Book fair

- **B. Practice your MI skills**: You volunteered for the book fair at your child's school, and they've asked you to make sure everyone wears a mask. A parent comes in without a mask on.

- **A. Misinformed (resistant)**: You don't understand why you should wear a mask at the school event – you heard they don't help stop COVID spread anyway.

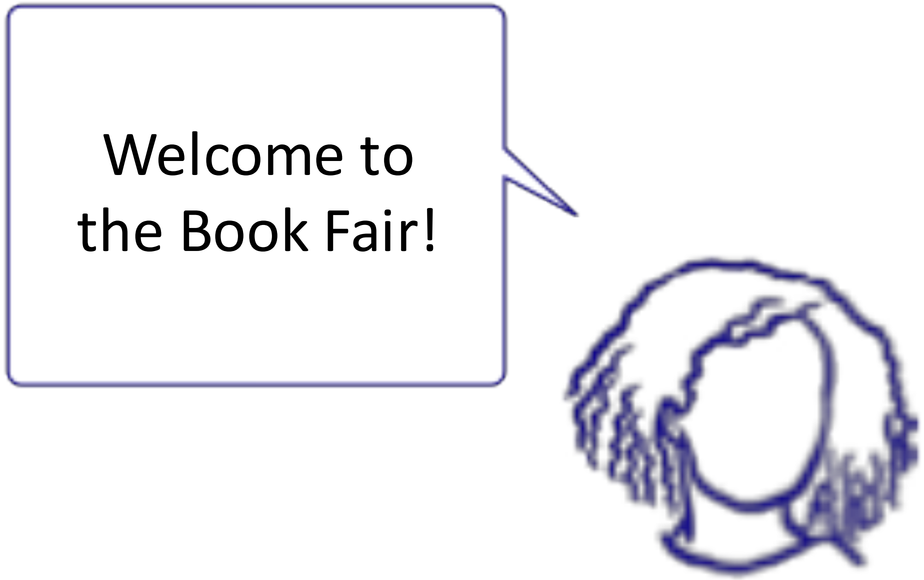

B. Practice your MI skills

Remember
O – Open ended questions
A – Affirmations
R – Reflective listening
S – Summaries

(Person A = Alphabetical by first name)

Image (5) from: https://www.hhs.gov/sites/default/files/health-misinformation-toolkit-english.pdf

M.I. Practice 2: Book fair

SWITCH!

- **A. Practice your MI skills**: You volunteered for the book fair at your child's school, and they've asked you to make sure everyone wears a mask. A parent comes in without a mask on.

- **B. Misinformed (resistant)**: You don't understand why you should wear a mask at the school event – you heard they don't help stop COVID spread anyway.

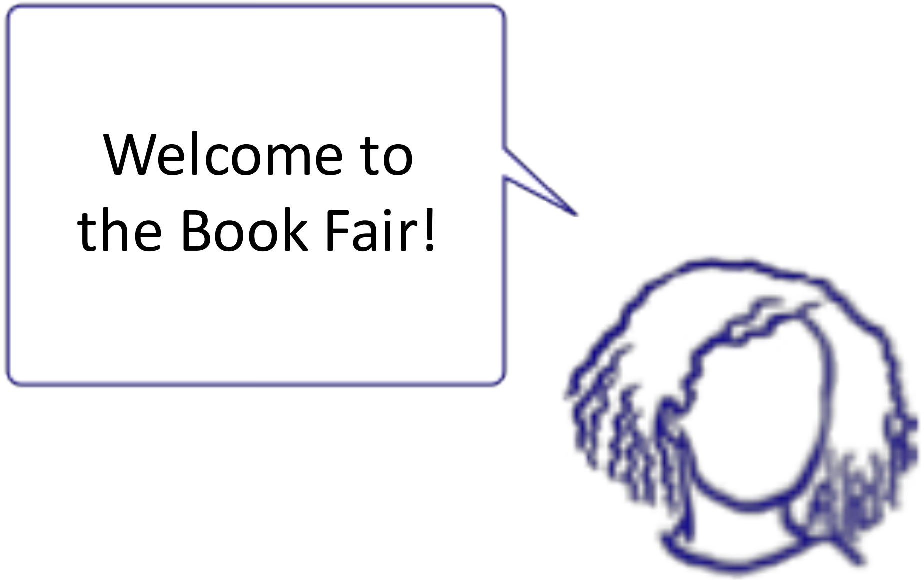

<u>Remember</u>

O – Open ended questions
A – Affirmations
R – Reflective listening
S – Summaries

A. Practice your MI skills

(Person A = Alphabetical by first name)

Image (5) from: https://www.hhs.gov/sites/default/files/health-misinformation-toolkit-english.pdf

Practice debrief

- What went well in this scenario?
- Were there things you struggled with?
- Things you found easier than expected?

Training Debrief

- What worked well?
- What would you change?
- What would be helpful to you to keep going?
  (e.g., more trainings, booster sessions, chat group, meet-ups)
- Other questions?

Thank you

Please take our short survey

- Check your email (the one you used in the pre-survey) for the link
- Once complete, you'll receive:
  - An email with a $20 gift certificate
  - An invitation to schedule a follow-up interview
  - Handouts from the training

Questions or concerns

Katie Cueva: kcueva@alaska.edu
Drew Cameron: drew.cameron@yale.edu

UNIVERSITY of ALASKA ANCHORAGE
ALASKA HEALTH MISINFORMATION RESPONSE PROJECT
Yale SCHOOL OF PUBLIC HEALTH

References

1) CDC COVID Data Tracker, https://covid.cdc.gov/covid-data-tracker/#datatracker-home, Accessed: July 2022.
2) World Health Organization, WHO COVID-19 Dashboard, https://data.who.int/dashboards/covid19/deaths, Accessed: July 2022.
3) CDC, National Center for Health Statistics. https://www.cdc.gov/nchs/fastats/leading-causes-of-death.htm. Accessed: July 2022.
4) CDC COVID-19. https://www.cdc.gov/covid/prevention/index.html, Accessed: July 2022.
5) CDC Respiratory Illnesses. About Physical Distancing and Respiratory Illnesses. https://www.cdc.gov/respiratory-viruses/prevention/physical-distancing.html. Accessed: July 2022
6) U.S. Department of Health and Human Services. Health Misinformation. https://www.hhs.gov/surgeongeneral/reports-and-publications/health-misinformation/index.html. Accessed: July 2022.
7) Inoculation Science. https://inoculation.science/. Accessed: July 2022
8) Surgeon General's Health Misinformation Toolkit. https://www.hhs.gov/sites/default/files/health-misinformation-toolkit-english.pdf, Accessed: July 2022

Images

1) https://www.fda.gov/emergency-preparedness-and-response/preparedness-research/cellular-signaling-and-immune-correlates-sars-cov-2-infection
2) https://nutrition.org/nutritional-epidemiologists-encounter-with-coronavirus-covid-19-in-wuhan-china/
3) Alaska Department of Health, July 2022
4) https://www.hhs.gov/surgeongeneral/reports-and-publications/health-misinformation/index.html
5) https://www.hhs.gov/sites/default/files/health-misinformation-toolkit-english.pdf

Motivational Interviewing

...an empathic, person-centered approach that prepares people for change by helping them resolve uncertainty, enhance internal motivation, and build confidence to change.

O.A.R.S.

Open ended questions

- Invite them to voice their concerns in their own words; *avoid yes or no questions*
- Don't lead them in a specific direction
- Be willing to listen to the WHOLE response

Affirmations

- Recognize their strengths
- Acknowledge positive change behaviors (no matter how large or small)
- Build confidence in a genuine and consistent way

Reflective Listening

This is the hardest part to do well!

- Repeat or rephrase their concerns
- ...or reflect on the feelings they evoked
- Your voice turns DOWN at the end of the sentence

Summaries

- Start by summarizing the conversation
- Give special attention to CHANGE STATEMENTS
- If they express uncertainty, include both sides
- Include concise objective information
- End with an invitation

Examples

- "Help me understand ..."
- "Please tell me more of your concerns..."
- "How would you like things to be different?"

- "I can see that you really care about your family's health and safety"
- "Its clear that you're very resourceful"
- "I've really enjoyed talking to you"

- "So, you feel concerned about ..."
- "It sounds like you want to know more about vaccine safety ... "
- "You're wondering if ... "

- **"Let me see if I understand so far...**
  - you want to protect your kids, but you're also worried about vaccine safety.
  - I've been following the vaccine trial - can I share some of what I've learned?
  - Did I miss anything?

Signs of Resistance

- **Interrupting**
  Talking over, cutting off
- **Arguing**
  Challenging, discounting, hostility
- **Ignoring**
  Inattention, non-response, sidetracking
- **Denying**
  Blaming, disagreeing, minimizing, reluctance

Strategies to handle resistance

- **Reflection**
  Acknowledge client's disagreement or emotion
- **Emphasize personal choice & control**
  Assure them THEY determine what happens
- **Clarification**
  Verify that you understand their perspective
- **Shift Focus**
  Talk about points of agreement

Announce **I**nquire **M**irror **S**upport

***AIMS** describes the general phases and goals of an encounter with a vaccine-hesitant individual.*

A.I.M.S	Examples
**Announce**<br>Start with a statement. Most patients (70%+) will accept a vaccine recommendation at the "Announce" stage of the conversation. If your client demonstrates hesitancy or resistance, then move on to the "Inquire" stage of the conversation.	• "Influenza season is just beginning, so we'll give you your flu shot at the end of today's visit."<br>• "I see Casey is 2yrs and due for her MMR vaccine. We will give that to her at the end of the visit today."
**Inquire**<br>Don't interrupt. Seek to identify and understand what their concerns are using open-ended questions. Try to get that information in a very neutral non-judgmental way .	• "May I ask why? What have you heard in your community?" "You seem undecided. What are your thoughts about this vaccine?"<br>• "Please tell me more about your concerns?"<br>• "You seem to have mixed feelings....would you tell me more...?"
**Mirror**<br>Restate/reflect to the person what you heard. Asking permission first can increase *receptivity*. This communicates that you heard their concerns and how they *feel* about them.	• "With your permission let's review your concerns so we can make sure I understand..."<br>• "If I understand you correctly, your friends shared___ about the dangers of vaccines. It sounds like you want to know more about vaccine safety ... Do I have that right?"
**Secure Trust**<br>Secure trust in the relationship and the opportunity to succeed another day. State you may disagree but respect their opinion and demonstrate mutual concern for the health of their child and themselves.	• "In my professional judgment vaccination is the best option but I respect your right to decline today. Let's talk more at your next visit. Meanwhile here's what do look for..."<br>• "Although I feel strongly that you are vaccinated today, I can see you still have concerns, here is some additional information.."
