# Supplementary material for: Building better conversations: results of a community-based online health misinformation and motivational interviewing training program in Alaska
Source: BMC Public Health. 2026 Apr 2;26:1542. doi: 10.1186/s12889-026-26611-1 (PMC13169531; doi:10.1186/s12889-026-26611-1)
Supplement: Supplementary file 2 — Supplementary Material 2. [file 12889_2026_26611_MOESM2_ESM.docx]

Pre- Community Adapted Trainings

Start of Block: Misinfo ID

Q1 <img src="https://uaa.co1.qualtrics.com/CP/Graphic.php?IM=IM_9mmLqZHuFsgOIya" style="width: 575px; height: 204px;" />

|  | NOT AT ALL - 1 | 2 | 3 | 4 | 5 | 6 | VERY - 7 |
| --- | --- | --- | --- | --- | --- | --- | --- |
| <strong>How manipulative do you find this post?</strong> |  |  |  |  |  |  |  |

Q2 <img src="https://uaa.co1.qualtrics.com/CP/Graphic.php?IM=IM_9tBhJceDQnXT7XE" style="width: 575px; height: 227px;" />

|  | NOT AT ALL - 1 | 2 | 3 | 4 | 5 | 6 | VERY - 7 |
| --- | --- | --- | --- | --- | --- | --- | --- |
| <strong>How manipulative do you find this post?</strong> |  |  |  |  |  |  |  |

Q3 <img src="https://uaa.co1.qualtrics.com/CP/Graphic.php?IM=IM_4VLcn36HATHY1Ia" style="width: 575px; height: 222px;" />

|  | NOT AT ALL - 1 | 2 | 3 | 4 | 5 | 6 | VERY - 7 |
| --- | --- | --- | --- | --- | --- | --- | --- |
| <strong>How manipulative do you find this post?</strong> |  |  |  |  |  |  |  |

Q4 <img src="https://uaa.co1.qualtrics.com/CP/Graphic.php?IM=IM_50idns5PRov1LIq" style="width: 575px; height: 223px;" />

|  | NOT AT ALL - 1 | 2 | 3 | 4 | 5 | 6 | VERY - 7 |
| --- | --- | --- | --- | --- | --- | --- | --- |
| <strong>How manipulative do you find this post?</strong> |  |  |  |  |  |  |  |

Q5 <img src="https://uaa.co1.qualtrics.com/CP/Graphic.php?IM=IM_0dYTXaqaurs1M2i" style="width: 575px; height: 227px;" />

|  | NOT AT ALL - 1 | 2 | 3 | 4 | 5 | 6 | VERY - 7 |
| --- | --- | --- | --- | --- | --- | --- | --- |
| <strong>How manipulative do you find this post?</strong> |  |  |  |  |  |  |  |

End of Block: Misinfo ID

Start of Block: Pre-survey

Q8.1 Please rate your level of confidence in your ability to do the following activities: (NO CONFIDENCE to VERY CONFIDENT)

|  | NO CONFIDENCE - 1 | 2 | 3 | 4 | 5 | 6 | 7 | 8 | 9 | VERY CONFIDENT - 10 |
| --- | --- | --- | --- | --- | --- | --- | --- | --- | --- | --- |
| Finding accurate COVID-19 related information |  |  |  |  |  |  |  |  |  |  |
| Detecting misinformation about COVID-19 |  |  |  |  |  |  |  |  |  |  |

| Page Break |  |
| --- | --- |

Q26 Please rate your level of confidence in your ability to do the following activities: (NO CONFIDENCE to VERY CONFIDENT)

|  | NO CONFIDENCE - 1 | 2 | 3 | 4 | 5 | 6 | 7 | 8 | 9 | VERY CONFIDENT - 10 |
| --- | --- | --- | --- | --- | --- | --- | --- | --- | --- | --- |
| Communicating with <strong>friends and family</strong> about COVID-19 |  |  |  |  |  |  |  |  |  |  |
| Communicating with <strong>customers, clients, or people other than</strong> <strong>friends and family</strong> about COVID-19 |  |  |  |  |  |  |  |  |  |  |

| Page Break |  |
| --- | --- |

Q19 What do you hope to learn by taking this training?

________________________________________________________________

________________________________________________________________

________________________________________________________________

________________________________________________________________

________________________________________________________________

End of Block: Pre-survey

Start of Block: Demographics

Q10 Do you identify as:

- Male
- Female
- Non-binary / third gender
- Prefer not to say

| 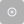 |
| --- |

Q11 In which region do you primarily reside?

- Aleutians East Borough
- Aleutians West Census Area
- Anchorage
- Bethel Census Area
- Bristol Bay Borough
- Chugach Census Area
- Copper River Census Area
- Denali Borough
- Dillingham Census Area
- Fairbanks North Star Borough
- Haines Borough
- Hoonah–Angoon Census Area
- Juneau
- Kenai Peninsula Borough
- Ketchikan Gateway Borough
- Kodiak Island Borough
- Kusilvak Census Area
- Lake and Peninsula Borough
- Matanuska-Susitna Borough
- Nome Census Area
- North Slope Borough
- Northwest Arctic Borough
- Petersburg Borough
- Prince of Wales-Hyder Census Area
- Sitka
- Skagway
- Southeast Fairbanks Census Area
- Wrangell
- Yakutat
- Yukon-Koyukuk Census Area
- non-Alaska U.S. state or territory
- Outside the U.S.

Q19 What is your vocation?

________________________________________________________________

Q13 Please describe your job title / profession in a few words:

________________________________________________________________

Q14 Please check the box or boxes that best describe your racial background:

- Alaska Native
- American Indian
- White
- Black
- Asian
- Hispanic/Latino
- Pacific Islander
- Other __________________________________________________

Q15 Please check the box that best describes your age:

- <18
- 18-20
- 21-29
- 30-39
- 40-49
- 50-59
- 60-69
- 70-79
- 80 or wiser

Q16 What is your highest level of formal education?

- Some high school or less
- High school diploma or GED
- Some college or university
- Associate degree
- Bachelor degree
- Master degree
- Doctoral degree

Q17 What is your approximate salary level?

- $10,000 per year or less
- $10,001-$20,000 per year
- $20,001-$30,000 per year
- $30,001-$40,000 per year
- $40,001-$50,000 per year
- $50,001-$60,000 per year
- $60,001-$70,000 per year
- $70,001-$80,000 per year
- $80,001-$90,000 per year
- $90,001-$100,000 per year
- $100,001 per year or more

Q18 Have you received an initial COVID-19 vaccination? <br>(Two doses of Pfizer/Biontech, or Moderna, OR one dose of Jansen/Johnson&Johnson)

- Yes
- No
- Only first dose of Moderna or Pfizer

| Page Break |  |
| --- | --- |

Display this question:

If Have you received an initial COVID-19 vaccination?  (Two doses of Pfizer/Biontech, or Moderna, OR... = Yes

Q19 Have you received a COVID-19 vaccination booster?

- Yes
- No

End of Block: Demographics

Start of Block: In case

Q32 At the end of the training, once we’ve asked a few more questions, we would like to give you a $20 Fred Meyer gift card as a thank you. Please indicate if you would like to get a gift card:

- Yes, I would like you to email me a gift card
- No, I would not like you to send me a gift card

Q31 In case we lose you before the training ends, may we follow-up with you via email after this training to ask you to join us for a short focus group or interview?

- Yes
- No

End of Block: In case

Post- Community Adapted Trainings

Start of Block: Misinfo ID

Conspiracy Theory <img src="https://uaa.co1.qualtrics.com/CP/Graphic.php?IM=IM_9mmLqZHuFsgOIya" style="width: 575px; height: 204px;" />

|  | NOT AT ALL - 1 | 2 | 3 | 4 | 5 | 6 | VERY - 7 |
| --- | --- | --- | --- | --- | --- | --- | --- |
| <strong>How manipulative do you find this post?</strong> |  |  |  |  |  |  |  |

Emotional Language <img src="https://uaa.co1.qualtrics.com/CP/Graphic.php?IM=IM_9tBhJceDQnXT7XE" style="width: 575px; height: 227px;" />

|  | NOT AT ALL - 1 | 2 | 3 | 4 | 5 | 6 | VERY - 7 |
| --- | --- | --- | --- | --- | --- | --- | --- |
| <strong>How manipulative do you find this post?</strong> |  |  |  |  |  |  |  |

Fake Experts <img src="https://uaa.co1.qualtrics.com/CP/Graphic.php?IM=IM_4VLcn36HATHY1Ia" style="width: 575px; height: 222px;" />

|  | NOT AT ALL - 1 | 2 | 3 | 4 | 5 | 6 | VERY - 7 |
| --- | --- | --- | --- | --- | --- | --- | --- |
| <strong>How manipulative do you find this post?</strong> |  |  |  |  |  |  |  |

False dichotomy <img src="https://uaa.co1.qualtrics.com/CP/Graphic.php?IM=IM_50idns5PRov1LIq" style="width: 575px; height: 223px;" />

|  | NOT AT ALL - 1 | 2 | 3 | 4 | 5 | 6 | VERY - 7 |
| --- | --- | --- | --- | --- | --- | --- | --- |
| <strong>How manipulative do you find this post?</strong> |  |  |  |  |  |  |  |

Control Ad <img src="https://uaa.co1.qualtrics.com/CP/Graphic.php?IM=IM_0dYTXaqaurs1M2i" style="width: 575px; height: 227px;" />

|  | NOT AT ALL - 1 | 2 | 3 | 4 | 5 | 6 | VERY - 7 |
| --- | --- | --- | --- | --- | --- | --- | --- |
| <strong>How manipulative do you find this post?</strong> |  |  |  |  |  |  |  |

End of Block: Misinfo ID

Start of Block: Post-survey

| 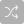 | 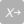 |
| --- | --- |

Q51 To the best of your knowledge which of these statements is FALSE?

- A COVID-19 vaccine is recommended if you've already had COVID-19.
- COVID-19 vaccines have been proven to reduce fertility.
- Washing hands regularly using disinfectants or soap and water prevents the spread of COVID-19.
- COVID-19 vaccines have been proven to be safe and highly effective.
- Masks reduce the spread of COVID-19.

| Page Break |  |
| --- | --- |

| 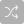 |
| --- |

Q1 “Misinformation” is defined as (select only one):

- Incorrect or misleading information that can be shared
- Unverified stories that can spread rapidly
- False information that is spread either by mistake or the intent to mislead
- I don’t know

| 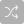 |
| --- |

Q48 Which of the following is NOT a skill to address misinformation?

- Listen
- Empathize
- Point to credible sources
- Fact-check specific statements to prove someone wrong
- Use inclusive language

| Page Break |  |
| --- | --- |

Q49 Motivational interviewing is an person-centered approach that prepares people for change by helping them resolve uncertainty, enhance internal motivation, and build confidence to change.

- True
- False

| 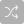 |
| --- |

Q50 Which of the following is not part of motivational interviews?

- “Yes” or “no” questions
- Offering affirmations
- Reflexive listening
- Summarizing the conversation

| Page Break |  |
| --- | --- |

Q33 Please rate your level of confidence in your ability to do the following activities: (NO CONFIDENCE to VERY CONFIDENT)

|  | NO CONFIDENCE - 1 | 2 | 3 | 4 | 5 | 6 | 7 | 8 | 9 | VERY CONFIDENT - 10 |
| --- | --- | --- | --- | --- | --- | --- | --- | --- | --- | --- |
| Finding accurate COVID-19 related information |  |  |  |  |  |  |  |  |  |  |
| Detecting misinformation about COVID-19 |  |  |  |  |  |  |  |  |  |  |

| Page Break |  |
| --- | --- |

Q35 Please rate your level of confidence in your ability to do the following activities: (NO CONFIDENCE to VERY CONFIDENT)

|  | NO CONFIDENCE - 1 | 2 | 3 | 4 | 5 | 6 | 7 | 8 | 9 | VERY CONFIDENT - 10 |
| --- | --- | --- | --- | --- | --- | --- | --- | --- | --- | --- |
| Communicating with <strong>friends and family</strong> about COVID-19 |  |  |  |  |  |  |  |  |  |  |
| Communicating with <strong>customers, clients, or people other than</strong> <strong>friends and family</strong> about COVID-19 |  |  |  |  |  |  |  |  |  |  |

End of Block: Post-survey

Start of Block: Personal outcomes

Q46 How would you rate each training module on the quality of <strong>presentation</strong> (POOR to EXCELLENT)?

|  | POOR PRESENTATION - 1 | 2 | 3 | 4 | 5 | 6 | 7 | 8 | 9 | EXCELLENT PRESENTATION - 10 |
| --- | --- | --- | --- | --- | --- | --- | --- | --- | --- | --- |
| Module 1 [<strong>COVID-19 in Alaska and Recommendations</strong>] |  |  |  |  |  |  |  |  |  |  |
| Module 2 [<strong>Types of Misinformation</strong>] |  |  |  |  |  |  |  |  |  |  |
| Module 3 [<strong>Conversations and Motivational Interviewing</strong>] |  |  |  |  |  |  |  |  |  |  |

Q47 How <strong>informative</strong> did you find each training module<strong> </strong>(NOT INFORMATIVE to EXTREMELY INFORMATIVE)?

|  | NOT INFORMATIVE - 1 | 2 | 3 | 4 | 5 | 6 | 7 | 8 | 9 | EXTREMELY INFORMATIVE - 10 |
| --- | --- | --- | --- | --- | --- | --- | --- | --- | --- | --- |
| Module 1 [<strong>COVID-19 in Alaska and Recommendations</strong>] |  |  |  |  |  |  |  |  |  |  |
| Module 2 [<strong>Types of Misinformation</strong>] |  |  |  |  |  |  |  |  |  |  |
| Module 3 [<strong>Conversations and Motivational Interviewing</strong>] |  |  |  |  |  |  |  |  |  |  |

| Page Break |  |
| --- | --- |

Q56 <b>Please take a few moments to reflect on the training...</b>

Q23 What did you like about this training?

________________________________________________________________

________________________________________________________________

________________________________________________________________

________________________________________________________________

________________________________________________________________

Q24 What could be improved about this training?

________________________________________________________________

________________________________________________________________

________________________________________________________________

________________________________________________________________

________________________________________________________________

Q25 Did you learn what you hoped to learn?

________________________________________________________________

________________________________________________________________

________________________________________________________________

________________________________________________________________

________________________________________________________________

| Page Break |  |
| --- | --- |

Q26 As a result of completing this training, I plan to…

|  | Yes | No | Don't know |
| --- | --- | --- | --- |
| …share facts presented in the training related to COVID prevention or treatment |  |  |  |
| …be more skeptical of COVID-related information I see |  |  |  |
| …use the OARS technique in COVID-related conversations. |  |  |  |

| Page Break |  |
| --- | --- |

Q57 As a result of completing this training, I plan to…

|  | Yes | No | Don't know |
| --- | --- | --- | --- |
| … be more likely to wear a mask when I’m indoors in public |  |  |  |
| …be more likely to physical distance from other people when out in public |  |  |  |
| …be more likely to avoid touching eyes, nose, mouth with unwashed hands |  |  |  |
| …encourage others to get vaccinated and boosted with more confidence |  |  |  |

Q28 Please tell us more...

________________________________________________________________

________________________________________________________________

________________________________________________________________

________________________________________________________________

________________________________________________________________

| Page Break |  |
| --- | --- |

Display this question:

If As a result of completing this training, I plan to… = …share facts presented in the training related to COVID prevention or treatment [ No ]

Q42 You stated that you <u>don't</u> plan to share facts related to COVID. What would make you more likely to share facts related to COVID?

________________________________________________________________

________________________________________________________________

________________________________________________________________

________________________________________________________________

________________________________________________________________

Display this question:

If As a result of completing this training, I plan to… = …use the OARS technique in COVID-related conversations. [ No ]

Q41 You stated that you <u>don't</u> plan to use the OARS technique in COVID-related conversations. What else would help you to apply this technique in your COVID-related conversations?

________________________________________________________________

________________________________________________________________

________________________________________________________________

________________________________________________________________

________________________________________________________________

| Page Break |  |
| --- | --- |

Q31 Is there anything else you would like to share with us about this training?

________________________________________________________________

________________________________________________________________

________________________________________________________________

________________________________________________________________

________________________________________________________________

End of Block: Personal outcomes

Start of Block: wrap-up

Q59 Thank you for your response! You are now eligible for a $20 gift certificate. <br><br>But wait, before you go... would you be open to signing up for a one-on-one interview or focus group?<br>

- Yes, help me sign up
- No, let's finish the survey

3mo- Community Adapted Trainings

Start of Block: Community Training Consent

Q84 This is the second post-survey for the <strong>Health Misinformation in Alaska: Building Better Conversations training</strong>. Please answer the questions to the best of your ability! The responses collected from the group will help us make our training better.

Start of Block: Misinfo ID

Conspiracy theory <img src="https://uaa.co1.qualtrics.com/CP/Graphic.php?IM=IM_9mmLqZHuFsgOIya" style="width: 575px; height: 204px;" />

|  | NOT AT ALL - 1 | 2 | 3 | 4 | 5 | 6 | VERY - 7 |
| --- | --- | --- | --- | --- | --- | --- | --- |
| <strong>How manipulative do you find this post?</strong> |  |  |  |  |  |  |  |

Emotional Language <img src="https://uaa.co1.qualtrics.com/CP/Graphic.php?IM=IM_9tBhJceDQnXT7XE" style="width: 575px; height: 227px;" />

|  | NOT AT ALL - 1 | 2 | 3 | 4 | 5 | 6 | VERY - 7 |
| --- | --- | --- | --- | --- | --- | --- | --- |
| <strong>How manipulative do you find this post?</strong> |  |  |  |  |  |  |  |

Fake Experts <img src="https://uaa.co1.qualtrics.com/CP/Graphic.php?IM=IM_4VLcn36HATHY1Ia" style="width: 575px; height: 222px;" />

|  | NOT AT ALL - 1 | 2 | 3 | 4 | 5 | 6 | VERY - 7 |
| --- | --- | --- | --- | --- | --- | --- | --- |
| <strong>How manipulative do you find this post?</strong> |  |  |  |  |  |  |  |

False Dicotomy <img src="https://uaa.co1.qualtrics.com/CP/Graphic.php?IM=IM_50idns5PRov1LIq" style="width: 575px; height: 223px;" />

|  | NOT AT ALL - 1 | 2 | 3 | 4 | 5 | 6 | VERY - 7 |
| --- | --- | --- | --- | --- | --- | --- | --- |
| <strong>How manipulative do you find this post?</strong> |  |  |  |  |  |  |  |

Control Ad <img src="https://uaa.co1.qualtrics.com/CP/Graphic.php?IM=IM_0dYTXaqaurs1M2i" style="width: 575px; height: 227px;" />

|  | NOT AT ALL - 1 | 2 | 3 | 4 | 5 | 6 | VERY - 7 |
| --- | --- | --- | --- | --- | --- | --- | --- |
| <strong>How manipulative do you find this post?</strong> |  |  |  |  |  |  |  |

End of Block: Misinfo ID

Start of Block: Post-survey

| 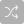 | 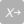 |
| --- | --- |

Q74 To the best of your knowledge which of these statements is FALSE?

- A COVID-19 vaccine is recommended if you've already had COVID-19.
- COVID-19 vaccines have been proven to reduce fertility.
- Washing hands regularly using disinfectants or soap and water prevents the spread of COVID-19.
- COVID-19 vaccines have been proven to be safe and highly effective.
- Masks reduce the spread of COVID-19.

| Page Break |  |
| --- | --- |

| 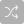 |
| --- |

Q76 “Misinformation” is defined as (select only one):

- Incorrect or misleading information that can be shared
- Unverified stories that can spread rapidly
- False information that is spread either by mistake or the intent to mislead
- I don’t know

| 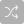 |
| --- |

Q78 Which of the following is not a skill to address misinformation?

- Listen
- Empathize
- Point to credible sources
- Fact-check specific statements to prove someone wrong
- Use inclusive language

| Page Break |  |
| --- | --- |

Q80 Motivational interviewing is a person-centered approach that prepares people for change by helping them resolve uncertainty, enhance internal motivation, and build confidence to change.

- True
- False

| 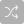 |
| --- |

Q79 Which of the following is not part of motivational interviews?

- “Yes” or “no” questions
- Offering affirmations
- Reflexive listening
- Summarizing the conversation

| Page Break |  |
| --- | --- |

Q86 Please rate your level of confidence in your ability to do the following activities: (NO CONFIDENCE to VERY CONFIDENT)

|  | NO CONFIDENCE - 1 | 2 | 3 | 4 | 5 | 6 | 7 | 8 | 9 | VERY CONFIDENT - 10 |
| --- | --- | --- | --- | --- | --- | --- | --- | --- | --- | --- |
| Finding accurate COVID-19 related information |  |  |  |  |  |  |  |  |  |  |
| Detecting misinformation about COVID-19 |  |  |  |  |  |  |  |  |  |  |

| Page Break |  |
| --- | --- |

Q84 Please rate your level of confidence in your ability to do the following activities: (NO CONFIDENCE to VERY CONFIDENT)

|  | NO CONFIDENCE - 1 | 2 | 3 | 4 | 5 | 6 | 7 | 8 | 9 | VERY CONFIDENT - 10 |
| --- | --- | --- | --- | --- | --- | --- | --- | --- | --- | --- |
| Communicating with <strong>friends and family</strong> about COVID-19 |  |  |  |  |  |  |  |  |  |  |
| Communicating with <strong>customers, clients, or people other than</strong> <strong>friends and family</strong> about COVID-19 |  |  |  |  |  |  |  |  |  |  |

End of Block: Post-survey

Start of Block: Intentions

Q32 Since completing the Building Better Conversations training, I have been…

|  | More often | Less often | No Change | Don't know |
| --- | --- | --- | --- | --- |
| … sharing facts related to current COVID-19 prevention and treatment recommendations |  |  |  |  |
| … aware of potential COVID-19 misinformation I might encounter |  |  |  |  |
| … aware of <em>other </em>potential misinformation I might encounter |  |  |  |  |
| …using Motivational Interviewing techniques (OARS) in COVID-19 related conversations |  |  |  |  |
| … using Motivational Interviewing techniques (OARS) in other conversations |  |  |  |  |

| Page Break |  |
| --- | --- |

Q89 Since completing the Building Better Conversations training, I have been…

|  | More often | Less often | No Change | Don't know |
| --- | --- | --- | --- | --- |
| … wearing a mask when I’m indoors in public |  |  |  |  |
| …physically distancing from other people when out in public |  |  |  |  |
| …avoiding touching eyes, nose, mouth with unwashed hands |  |  |  |  |
| …encouraging others to get vaccinated and boosted with more confidence |  |  |  |  |

Q33 Please tell us more...

________________________________________________________________

________________________________________________________________

________________________________________________________________

________________________________________________________________

________________________________________________________________

End of Block: Intentions

Start of Block: Implementation questions

Q37 Have you tried to use any of the motivational interviewing (OARS) techniques from the training with other people in your personal or professional life?

- Yes
- No
- Don't know

| Page Break |  |
| --- | --- |

Display this question:

If Have you tried to use any of the motivational interviewing (OARS) techniques from the training wi... = Yes

Q39 Tell us more -- what was one scenario and how did it go?

________________________________________________________________

________________________________________________________________

________________________________________________________________

________________________________________________________________

________________________________________________________________

Display this question:

If Have you tried to use any of the motivational interviewing (OARS) techniques from the training wi... = Yes

Q92 How would you describe your relationship(s) after the conversation?

- Better than before
- About the same
- Worse than before

Display this question:

If Have you tried to use any of the motivational interviewing (OARS) techniques from the training wi... = Yes

Q93 As a result of the Building Better Conversations training do you think this conversation went...

- Better than it could have
- About the same as it would have
- Worse than otherwise

Display this question:

If Have you tried to use any of the motivational interviewing (OARS) techniques from the training wi... = No

Or Have you tried to use any of the motivational interviewing (OARS) techniques from the training wi... = Don't know

Q58 Is there a particular reason you have not been able to use these techniques?

________________________________________________________________

________________________________________________________________

________________________________________________________________

________________________________________________________________

________________________________________________________________

| Page Break |  |
| --- | --- |

stories Have you heard stories about others who took the training using any of the motivational interviewing techniques?

- Yes
- No
- Don't know

| Page Break |  |
| --- | --- |

Display this question:

If Have you heard stories about others who took the training using any of the motivational interview... = Yes

Q40 Briefly, what was one story? What happened?

________________________________________________________________

________________________________________________________________

________________________________________________________________

________________________________________________________________

________________________________________________________________

| Page Break |  |
| --- | --- |

Q41 In the last few months, have you shared anything you learned from this training with (select all that apply):

- Family members
- Friends
- Co-workers
- Patients
- Clients/Customers
- Neighbors
- Other community members
- ⊗Nobody
- Other __________________________________________________

| Page Break |  |
| --- | --- |

Display this question:

If If In the last few months, have you shared anything you learned from this training with (select all that apply): q://QID41/SelectedChoicesCount Is Greater Than 0

And In the last few months, have you shared anything you learned from this training with (select all... != Nobody

Q42 Please describe (that experience / those experiences):

________________________________________________________________

________________________________________________________________

________________________________________________________________

________________________________________________________________

________________________________________________________________

Display this question:

If In the last few months, have you shared anything you learned from this training with (select all... = Nobody

Q91 Why haven't you been able to share what you learned with anyone?

________________________________________________________________

________________________________________________________________

________________________________________________________________

________________________________________________________________

________________________________________________________________

| Page Break |  |
| --- | --- |

Q59 Would you be interested in a refresher training on Building Better Conversations?

- Yes
- No

| Page Break |  |
| --- | --- |

Display this question:

If Would you be interested in a refresher training on Building Better Conversations? = Yes

Q60 What topics would you want covered in a refresher training?

________________________________________________________________

________________________________________________________________

________________________________________________________________

________________________________________________________________

________________________________________________________________

| Page Break |  |
| --- | --- |

Display this question:

If Would you be interested in a refresher training on Building Better Conversations? = Yes

Q62 Are there any additional areas you would like to be covered?

________________________________________________________________

________________________________________________________________

________________________________________________________________

________________________________________________________________

________________________________________________________________

| Page Break |  |
| --- | --- |

Display this question:

If Would you be interested in a refresher training on Building Better Conversations? = Yes

Q61 How long after the initial training should a refresher training be offered?

________________________________________________________________

| Page Break |  |
| --- | --- |

Q63 Is there anything else that would help you to have Better Conversations about health misinformation and/or vaccine hesitancy?

________________________________________________________________

________________________________________________________________

________________________________________________________________

________________________________________________________________

________________________________________________________________

End of Block: Implementation questions
