# Supplementary material for: Building better conversations: results of a community-based online health misinformation and motivational interviewing training program in Alaska
Source: BMC Public Health. 2026 Apr 2;26:1542. doi: 10.1186/s12889-026-26611-1 (PMC13169531; doi:10.1186/s12889-026-26611-1)
Supplement: Supplementary file 3 — Supplementary Material 3. [file 12889_2026_26611_MOESM3_ESM.docx]

**Appendix 3**Cameron et al., *BMC Public Health*

**Post-Training Interview Guide**

**BBC Training Discussion Guide**

1. **Informed consent doc (share PDF and read first page)**
2. **Ask if it’s okay to record the interview**
3. **START RECORDING**

Main Research Question: *How acceptable, appropriate, and feasible are lay community trainings on misinformation identification and motivational interviewing skills for participants?*

Questions (with probing)

**Calibration**

1. Let’s start by having you describe what you do at your organization…
   - What is your formal title?
   - How long have you worked in your current position?
   - What are your formal responsibilities? Can you describe a typical day for you?
     1. How does your job require interaction with the public?
     2. How does your job involve public health topics? What topics?
     3. How often do you encounter misinformation in your professional life? Your personal life?

**Implementation Questions***Source*

1. Why did you want to take this training (how’d you learn about it)?
   - What made you decide to attend the training?
   - After attending the training, how do you think the skills you learned would be helpful to prepare you for an unexpected event/tough conversations about misinformation?

*Individual Self-efficacy; adaptation; KAB*

1. What parts of the training helped you to feel more confident in identifying misinformation?

Probing

- - What was your understanding of misinformation before you took this training? How has it changed?
  - Could you share some examples of times you’ve encountered misinformation?
    1. How much of this centered around COVID vaccines in particular?
  - What would you add to the training to help you feel more confident?
  - What would you take away?

1. What parts of the training helped you to feel more comfortable in talking to others (MI) about misinformation and vaccine hesitancy?

Probing

- - Were you familiar with motivational interviewing (or any of the MI skills we covered) before this training?
    - Could you share any examples of times you’ve used these skills?
      - Did any of these conversations center around vaccines?
    - Can you think of any examples of times you COULD have used these skills but didn’t? Why not?
  - What would you add to the training to help you feel more comfortable?
  - What would you take away?
  - Part of OARS that was more or less difficult to apply
    - In your professional life?
    - In your personal life?
  - Are there any skills you would want to practice more?

1. How do you feel about using elements of this training in the future?

Probing

- - In your professional life?
  - In your personal life?
  - Do you have feelings of anticipation, stress, enthusiasm?
  - Why? / Why not?
  - , or practice any new skills?
  - Are there any topics in particular? Vaccines?

1. Who do you think this training is useful for?
   - Are there particular group or individuals with whom you’d like to share what you’ve learned
   - Are there particular groups or individuals with whom you’d like to practice some of the techniques you’ve learned?
2. Is there anything else that you would like to share with me that I didn’t get to ask?

END RECORDING

1. ***Thanks very much for taking the time to talk with me today. We’re hoping that this training will be helpful to you going forward and that your experience will help us to improve it for other folks as well.

   We will be sending a follow-up survey to you by email in about 3 months and offering a $20 gift certificate for filling it out.

   We’re also hoping to follow up with folks in an interview to see how things have been going, and I’d like to talk to you again. Would you have any interest in chatting again in 3 months?***

*[if yes, or ambiguous]* ***I’ll be sending a thank you email within the next 24 hours that will include details on scheduling a follow-up in a few months.*** *[if yes]* ***Or, if you have your calendar open, we could pencil something in now…***

*[if no]* ***No problem. I really enjoyed talking to you today. Thank you again for taking the time out of your schedule to help us out.***

**Follow-up Interview Discussion Guide**

**3-Month BBC Training Discussion Guide
(FGDs and Interviews)**

1. **Informed consent doc (share PDF and read first page)**
2. **Ask if it’s okay to record the interview**
3. **START RECORDING**

Main Research Question:

*How successful has the reach and sustainability of the training program been (i.e., how well does respondent recall the training, how well have elements of the training been incorporated into daily practice, and which communities or groups has the respondent been able to engage with using elements of the training)?*

Discussion Questions (with probing)

**In the last three months …**

1. If we didn’t get a chance to talk before:
   - Could you remind me a little about your current position, where you are, what you do? What a typical day looks like for you?
2. What experiences have you had putting any of the training into practice in your personal or professional life?

Probing Questions:

- - Have you heard any COVID-19 misinformation in the last 3 months (in person or online)?
    - Where? Who? What was the experience like?
    - Did you respond? What was it like?
  - Have you used / referenced any of the training materials (handout / ppt)?
  - Have you applied any of the motivational interviewing techniques to address this misinformation?
    - In your professional life?
    - In your personal life?
      - What was your experience like?
      - How did folks respond?
  - Have you encountered any other kinds of misinformation in the last few months?
    - In local or state politics? – *Transferability of skills*

1. What parts of the training have stuck with you (MI, OARS, identifying misinformation, online resources)?

Probing

- - Are there skills you are more comfortable using? Why?
  - Are there skills you are less comfortable using? Why?
  - How often do you use any of the MI skills from the training?

1. What topic areas (or practices) from the training would be good to review?

Probing

- - Would you be interested in a refresher training?
    1. Would a refresher training be useful?
  - What would you want it to include?
  - What kind of other review would be useful?
  - Any other suggestions?

1. With whom have you shared any information from the training?

Probing

- - Have you shared any of the training materials with friends / family / coworkers / others?
    - What was their reaction?

1. With whom have you applied any of the lessons from the training?

Probing

- - Friends? Family? Co-workers? Neighbors? Clients/patients?
  - What other communities, groups or individuals have you tried reaching with lessons from the training?
    1. What communities / groups / individuals have you had a hard time reaching?
    2. Why do you think that is?

1. Do you plan to use any skills you learned in the training in the future?
   - If yes, how so? Where? With whom?
   - Misinformation skills?
   - Motivational interviewing skills?
2. In what other ways could we help you to find more support in putting lessons you learned from these trainings into practice?
3. Anything else you’d like to share with me?

**Note to interviewer: Motivational interviewing is a tool for building and maintaining relationships (primarily) and for moving people towards positive behavior change (secondarily). The elements of MI that respondents were exposed to include the OARS acronym: Open ended questions; Affirmations; Reflective Listening; and Summarizing.**

**Post Training Interview Code Book**

| **Meta Category** | **Parent Code** | **Baby Code** |
| --- | --- | --- |
| **Professional Life** | Interaction with the public | External population/organizational communications |
|  |  | Internal population/organizational communications |
|  |  | 1:1 interactions (external: client, patient, etc.) |
|  |  | 1:1 interactions (internal: HR, etc.) |
|  |  | No interaction with the public |
|  | Professional involvement with COVID | Creating COVID messages/guidelines/policies |
|  |  | Disseminating COVID messages/guidelines/policies |
|  |  | Implementing COVID guidelines/policies |
|  |  | Treating / preventing COVID |
|  |  | No professional involvement with COVID |
| **Experience with COVID Misinformation** | Examples of Misinformation encountered | About Covid treatments |
|  |  | About masks |
|  |  | About mistrust of government |
|  |  | about the Sars-CoV-2 virus |
|  |  | about vaccines |
|  |  | general- confusion about conflicting guidelines |
|  |  | non-covid misinformation |
|  | Experiences interacting with misinformed people | negative |
|  |  | neutral |
|  |  | no experience |
|  |  | positive |
|  | How would you have changed the interaction | don't know |
|  |  | would have done something else differently |
|  |  | would have used a technique from the training |
|  |  | would not have changed interaction |
|  | Misinformation in personal life | Family |
|  |  | friends |
|  |  | media (social media, internet, radio, tv, etc) |
|  |  | neighbors |
|  |  | none |
|  |  | other (in public interactions) |
|  | Misinformation in professional life | external (public, clients, patients) |
|  |  | none |
|  |  | within organization (co-workers, peers) |
|  |  | within organization (leadership, top-down) |
|  |  | within organization (subordinants, students) |
|  | Response to misinformation in personal life | general terms (avoid interaction) |
|  |  | general terms (engaged) |
|  |  | general terms (gave up on trying) |
|  |  | specific examples (avoids interaction) |
|  |  | specific examples (engaged) |
|  |  | specific examples (gave up on trying) |
|  | Response to misinformation in professional life | general terms (avoid interaction) |
|  |  | general terms (engaged) |
|  |  | general terms (gave up on trying) |
|  |  | specific examples (avoids interaction) |
|  |  | specific examples (engaged) |
|  |  | specific examples (gave up on trying) |
| **Prior Knowledge** | Prior skills in identifying misinformation | None |
|  |  | General understanding |
|  |  | Strong understanding |
|  |  | Specific training |
|  | Prior skills with MI before training | formal training or professional experience |
|  |  | have used technique(s) |
|  |  | none |
|  |  | related training or experience |
| **Training** | Challenges faced | challenges with format |
|  |  | challenges with the material |
|  |  | no challenges |
|  |  | technology |
|  |  | unforseen, external challenges |
|  | change in confidence, ability to recognize misinformation | decreased confidence |
|  |  | increased confidence |
|  |  | no change |
|  | general feelings towards BBC training | confused |
|  |  | negative |
|  |  | positive |
|  |  | unsure |
|  | how much did they learn or did it help | already applying lessons |
|  |  | gained new information |
|  |  | learned new ways of applying previous knowledge |
|  |  | no change |
|  |  | Had previous knowledge reinforced |
|  |  | plans to apply training |
|  | reasons to attend BBC training | don't know, no reason |
|  |  | emotional reasons |
|  |  | general curiosity, support |
|  |  | required training |
|  |  | social pressure, personal connection to training |
|  |  | wanted new skills |
|  | suggested areas of improvement | add something new |
|  |  | modify an existing component |
|  |  | modify the overall format |
|  |  | no suggestions |
|  |  | take something away |
|  | what participant DISLIKED about the training | COVID-19 recommendations module |
|  |  | general strcuture, organization, format |
|  |  | MI module |
|  |  | Misinformation module |
|  |  | other resources provided |
|  | what participant LIKED about the training | COVID-19 recommendations module |
|  |  | general structure, organization, format |
|  |  | MI module |
|  |  | Misinformation module |
|  |  | other resources provided |
| **Outlook for future (Reach)** | Future Anticipation | Negative |
|  |  | Neutral |
|  |  | Positive |
|  |  | Uncertain |
|  | Groups respondent wants to apply training materials | family |
|  |  | friends |
|  |  | general public |
|  |  | neighbors, community, acquaintances |
|  |  | specific groups |
|  | Groups respondent wants to share training materials | family |
|  |  | friends |
|  |  | general public |
|  |  | neighbors, community, acquaintances |
|  |  | specific groups |

**Follow-up Interview Codebook**

| **Meta Category** | **Parent Code** | **Baby Code** |
| --- | --- | --- |
| **Experiences Applying BBC Training (Misinformation Recognition)** | Identifying COVID Misinformation | None |
|  |  | **COVID isn't talked about anymore** |
|  |  | About Covid treatments |
|  |  | About masks |
|  |  | About mistrust of government |
|  |  | about the Sars-CoV-2 virus |
|  |  | about vaccines |
|  |  | general- confusion about conflicting guidelines |
|  |  | non-covid misinformation |
|  | Where did you identify COVID misinformation (personal life)? | Family |
|  |  | friends |
|  |  | media (social media, internet, radio, tv, etc) |
|  |  | neighbors |
|  |  | none |
|  |  | other (in public interactions) |
|  | Where did you identify COVID misinformation (professional life)? | external (public, clients, patients) |
|  |  | none |
|  |  | within organization (co-workers, peers) |
|  |  | within organization (leadership, top-down) |
|  |  | within organization (subordinants, students) |
| **Experiences Applying BBC Training (Motivational Interviewing)** | Used MI skills | None |
|  |  | Applied specific MI skills |
|  |  | Applied general MI concepts |
|  | Used MI in personal life | Family |
|  |  | friends |
|  |  | media (social media, internet, radio, tv, etc) |
|  |  | neighbors |
|  |  | none |
|  |  | other (in public interactions) |
|  |  | Specific groups |
|  | Used MI in professional life | external (public, clients, patients) |
|  |  | none |
|  |  | within organization (co-workers, peers) |
|  |  | within organization (leadership, top-down) |
|  |  | within organization (subordinants, students) |
|  |  | Specific groups |
|  | Experience using MI was... | negative |
|  |  | neutral |
|  |  | no experience |
|  |  | positive |
|  | Used MI in response to | COVID-19 misinformation |
|  |  | Other heatlh misinformation |
|  |  | Other non-health misinformation |
|  |  | Not in response to misinformation |
|  |  | **De-escalation of (potential) confrontations** |
|  |  | **Other contexts (generalized)** |
|  |  | Unprompted |
|  | Shared MI skills | none |
|  |  | family |
|  |  | friends |
|  |  | general public |
|  |  | neighbors, community, acquaintances |
|  |  | specific groups |
| **Sustainability / Outlook** | Experience recalling training | Had difficulty (general) |
|  |  | Did not have difficulty (general) |
|  |  | Specific things you remembered well |
|  |  | Specific things you had difficulty remembering |
|  | Frequency of use | not at all |
|  |  | infrequently |
|  |  | frequently |
|  | Refresher training | Would not want a refresher |
|  |  | Would want a refresher (general) |
|  |  | Would want refresher on specific material |
|  |  | Would want inclusion of new material or topics |
|  |  | Would want a different format of refresher |
|  |  | Open suggestions |
|  | Plans to use in future | Yes, plan to use skills myself in future |
|  |  | Yes, plan to share with others in the future |
|  |  | No, don't plan to use or share in future |
|  |  | Not sure |
|  | Groups to engage in the future | none |
|  |  | family |
|  |  | friends |
|  |  | general public |
|  |  | neighbors, community, acquaintances |
|  |  | specific groups |
|  | Improvements to original training | add something new |
|  |  | modify an existing component |
|  |  | modify the overall format |
|  |  | no suggestions |
|  |  | take something away |
